# Neutrophil Dynamics in Response to Cancer Therapies

**DOI:** 10.3390/cancers17152593

**Published:** 2025-08-07

**Authors:** Huazhen Xu, Xiaojun Chen, Yuqing Lu, Nihao Sun, Karis E. Weisgerber, Manzhu Xu, Ren-Yuan Bai

**Affiliations:** 1 Kennedy Krieger Institute, 707 N Broadway, Lab 520, Baltimore, MD 21205, USA; hxu99@jh.edu (H.X.); xchen277@jh.edu (X.C.); ylu146@jh.edu (Y.L.); kweisge1@jhu.edu (K.E.W.); mxu62@jh.edu (M.X.); 2 Department of Neurosurgery, Johns Hopkins University School of Medicine, 707 N Broadway, Lab 520, Baltimore, MD 21205, USA; 3 Department of Biomedical Engineering, Johns Hopkins University School of Medicine, 733 N Broadway, Baltimore, MD 21205, USA; nsun5@jh.edu

**Keywords:** neutrophils, tumor microenvironment, N1 neutrophils, N2 neutrophils, tumor-associated neutrophils, neutrophil polarization, cancer immunology, cancer therapy resistance, chemotherapy, radiotherapy, immunotherapy, oncolytic virotherapy, oncolytic bacterial therapy, innate immunity, inflammation and cancer

## Abstract

Neutrophils are increasingly recognized as key players in the tumor microenvironment (TME), displaying functional plasticity that enables them to either promote or inhibit cancer progression. Depending on environmental cues, tumor-associated neutrophils (TANs) may polarize toward antitumor “N1” or protumor “N2” phenotypes, exerting diverse effects on tumor growth, metastasis, immune modulation, and treatment response. While previous studies have focused on the pathological roles of TANs in cancer, less attention has been given to how cancer therapies themselves influence the behavior of TANs. This review provides a comprehensive synthesis of current knowledge regarding the dynamics of TANs in response to major cancer treatment modalities, including chemotherapy, radiotherapy, cell-based immunotherapies, and oncolytic viral and bacterial therapies. We discuss how these therapies influence TAN recruitment, polarization, and effector functions within the TME, and highlight key molecular regulators involved. By consolidating mechanistic and translational insights, this review emphasizes the potential to therapeutically reprogram TANs to enhance treatment efficacy. A deeper understanding of context-dependent TAN roles will be essential for developing more effective, neutrophil-informed cancer therapies.

## Simple Summary

Neutrophils are a type of white blood cell best known for fighting infections, but recent studies show they also play complex roles in cancer. Within tumors, neutrophils can adopt opposing identities: the “N1” type combats cancer by killing tumor cells and stimulating immune activity, while the “N2” type does the opposite—supporting tumor growth, blocking immune responses, and aiding disease progression. In this review, we explore how neutrophils respond to different cancer treatments, including chemotherapy, radiotherapy, immune cell therapy, and therapies using viruses or bacteria. We highlight how different therapeutic environments can drive neutrophils toward either the beneficial N1 or the harmful N2 state. Understanding what determines this polarization is key to improving outcomes. By understanding when neutrophils act in favor of or against treatment, scientists can design better strategies to fight cancer. This knowledge could lead to new therapies that guide neutrophils to support the immune system, reduce tumor spread, and improve the efficacy of current cancer treatments.

## 1. Introduction

Constituting 50–70% of circulating leukocytes [1,2], neutrophils are traditionally recognized as innate immune granulocytes but have more recently been identified as significant components of the tumor microenvironment (TME) [3,4,5]. Clinical observations consistently associate substantial neutrophil infiltration within tumors with enhanced disease progression and unfavorable patient prognosis [6]. These pro-tumoral effects are mediated through multiple mechanisms, including angiogenesis stimulation via Vascular Endothelial Growth Factor (VEGF) secretion, facilitation of tumor cell migration, and immune suppression through the release of neutrophil elastase (NE), interleukin 8 (IL-8), and arginase-1 (ARG1) [7,8,9,10,11,12]. Such tumor-promoting neutrophils, often classified as “N2” tumor-associated neutrophils (TANs), further support cancer progression by forming neutrophil extracellular traps (NETs), secreting pro-inflammatory cytokines such as IL-17 and tumor necrosis factor alpha (TNF-α), remodeling ECM components, and inhibiting T-cell-mediated immunity [13,14,15].

Conversely, neutrophils can also exhibit potent antitumor properties, commonly referred to as “N1” neutrophils. Their tumoricidal capacity involves reactive oxygen species (ROS) generation, cytotoxic enzyme release, antibody-dependent cellular cytotoxicity (ADCC), and activation of lymphocyte populations including cytotoxic T lymphocytes (CTLs) and natural killer (NK) cells [16,17,18]. These antitumoral “N1” neutrophils directly eliminate malignant cells through multiple effector mechanisms such as ROS and TNF-Related apoptosis-inducing ligand (TRAIL), while simultaneously enhancing adaptive immune responses [19,20,21].

The functional polarization of TANs exists along a continuum between these opposing phenotypes, with their behavior dynamically regulated by tumor microenvironmental cues [22]. Key regulatory factors include transforming growth factor-β (TGF-β), which promotes immunosuppressive N2 polarization, and type I interferons (IFN-β) that induce antitumoral N1 characteristics [23,24,25,26]. This phenotypic plasticity emphasizes the dual nature of neutrophils in cancer pathogenesis and illustrates the importance of understanding how therapeutic interventions influence neutrophil behavior, given their capacity to either compromise or potentiate treatment efficacy [27,28].

In summary, neutrophils in the TME are a double-edged sword, capable of promoting tumor growth and immune evasion, yet also potentially executing cytotoxic and immunostimulatory functions. While numerous reviews have summarized the tumor-promoting and tumor-suppressive functions of TANs, relatively few have examined how different cancer therapies modulate neutrophil phenotypes and functions, and what consequences these changes hold for therapeutic efficacy and resistance. The following sections review how neutrophil dynamics change in response to major cancer therapies—chemotherapy, radiotherapy (RT), cell-based immunotherapies, and oncolytic virotherapy (OVT)—integrating evidence from clinical studies on animal models. Where possible, we highlight emerging hypotheses and strategies to exploit or tame neutrophils for better therapeutic outcomes.

## 2. Polarization and Function Roles of TANs

**Figure 1:**
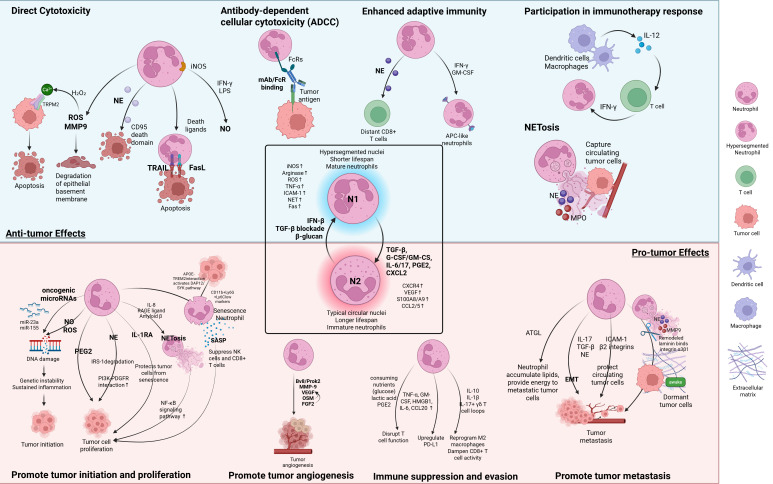
Polarization and Functional Diversity of TANs. TANs exhibit remarkable plasticity and polarize into either antitumor N1 or protumor N2 phenotypes in response to cues from the TME. N1 TANs are characterized by mature morphology and a hypersegmented nucleus, and mediate tumor suppression via direct cytotoxicity, ADCC, enhanced adaptive immunity, immunotherapy support, and NET-mediated tumor cell killing. In contrast, N2 TANs display immature morphology and promote tumor progression through the secretion of angiogenic and immunosuppressive factors, facilitation of immune evasion, and support of metastasis and tumor proliferation. Key polarization stimuli and effector mechanisms are depicted.

TANs exhibit remarkable functional plasticity, polarizing into either an anti-tumorigenic (N1) or pro-tumorigenic (N2) phenotype in response to cues from the TME. N1 TANs display hypersegmented nuclei and mature morphology [29,30], and are typically induced by IFN-β [26], blockade of TGF-β signaling [23], or β-glucan stimulation [31]. They are associated with enhanced cytotoxic activity, immunostimulatory functions, and a shorter lifespan [32,33]. Moreover, N1 TANs upregulate inducible nitric oxide synthase (iNOS), TNF-α, intercellular adhesion molecule-1 (ICAM-1), and Fas, while expressing lower levels of ARG1 and VEGF [23,26,30]. They also exhibit increased production of ROS, nitric oxide (NO) [34], and NETs [26]. Importantly, N1 TANs enhance CD8+ T cell recruitment and activation through the secretion of chemokines such as C-C motif chemokine ligand 3 (CCL3), C-X-C motif chemokine ligands 9 and 10 (CXCL9, CXCL10) [35], as well as proinflammatory cytokines including IL-12, TNF-α, and granulocyte-macrophage colony-stimulating factor (GM-CSF), thereby promoting anti-tumor immunity (Figure 1) [23,34].

In contrast, N2 TANs possess regular or circular nuclei and are induced by a wide array of tumor- and stroma-derived factors, including TGF-β, IL-6, IL-17, CXCL2, prostaglandin E2 (PGE2), granulocyte colony-stimulating factor (G-CSF), GM-CSF, and hyaluronic acid (HA) fragments [23,29,36,37,38,39,40]. These cells are characterized by an immature morphology, extended lifespan, and an immunosuppressive phenotype [29,30]. N2 TANs exhibit high expression of ARG1, along with low levels of iNOS, TNF-α, ICAM-1, and reduced NET formation [23,26,30]. They also secrete chemokines such as C-X-C Motif Chemokine Receptor 4 (CXCR4), CCL2, and CCL5, as well as VEGF and S100 calcium-binding proteins A8/A9 (S100A8/A9), which facilitate tumor progression, angiogenesis, and the recruitment of other immunosuppressive cells [23,29,41,42]. As aforementioned, Type I interferons, particularly IFN-β, are critical regulators of N1 polarization [26]. Mice deficient in IFN-β develop predominantly N2-like TANs, whereas melanoma patients treated with type I IFN therapy exhibit a shift toward N1-like neutrophil profiles, underscoring the relevance of these mechanisms in both murine models and human cancer (Figure 1) [26].

### 2.1. Anti-Tumor Functions of TANs

N1 TANs mediate anti-tumor effects through direct cytotoxicity, ADCC, enhancement of adaptive immunity, improved responses to immunotherapy, and NET-mediated tumor cell killing (Figure 1; Table 1) [41].

For direct cytotoxicity, N1 TANs release ROS and matrix metalloproteinases 9 (MMP-9), which degrade the epithelial basement membrane, dismantling tumor support structures [43]. Particularly, hydrogen peroxide (H_2_O_2_) secreted by TANs induces tumor apoptosis by triggering Ca^2+^ influx via the TRPM2 channel (transient receptor potential cation channel, subfamily M, member 2) [44]. Moreover, β-glucan–trained neutrophils produce elevated ROS for enhanced cytotoxic activity [31], and NE cleaves the CD95 death domain to selectively kill tumor cells [45]. MET-activated neutrophils are stimulated by TNF-α and its ligand hepatocyte growth factor (HGF). These cells generate NO via iNOS, which suppresses tumor growth and metastasis [46]. Additionally, N1 TANs express death ligands TRAIL and FasL, whose expression can be enhanced by IL-17 to promote apoptosis in tumor cells [20,40,47].

In the context of ADCC, Fc receptors on neutrophils bind the Fc region of tumor-targeting monoclonal antibodies (mAb), initiating antibody-mediated cytotoxicity against tumor cells [48]. Neutrophils also facilitate adaptive immune responses by enhancing CD8^+^ T cell activity via NE [45], cooperating with T cells through iNOS-mediated pathways [49], and differentiating into antigen-presenting neutrophils under the influence of IFN-γ and GM-CSF. These antigen-presenting neutrophils can capture tumor antigens, migrate to tumor-draining lymph nodes, and present antigens to activate T cells, initiating immune responses [50,51].

N1 TANs also enhance responses to immunotherapy through cytokine feedback loops. IL-12 from dendritic cells and macrophages activates T cells to secrete IFN-γ, which induces interferon regulatory factor (IRF1) in neutrophils, boosting their anti-tumor activity and reinforcing IL-12 production by macrophages [52,53]. Additionally, NETs immobilize circulating tumor cells (CTCs) via β1-integrin interactions and deliver cytotoxic proteins such as myeloperoxidase (MPO), NE, and MMPs to disrupt tumor membranes and prevent metastasis [54].

**Table 1:** Functional Mechanisms of N1 and N2 Neutrophils in the TME.

Neutrophil Phenotype	Function/ Tumor State Categories	Effector	Mechanism	References
N1	Direct Cytotoxicity	ROS, MMP9	TANs release ROS and MMP-9, degrading the epithelial basement membrane and inducing apoptosis via H_2_O_2_-triggered Ca^2+^ influx via TRPM2 channels.	[31,43,44]
		NE	NE secreted by TANs cleaves CD95 death domain, selectively killing tumor cells.	[45]
		NO	HGF and TNF-α activate MET. MET-activated TANs produce NO to inhibit tumor proliferation and metastasis.	[46]
		Death Ligands (TRAIL, FasL)	TANs expressing TRAIL and FasL induce apoptosis in tumor cells via death receptor signaling, enhanced by IL-17.	[20,40,47]
	ADCC	FcRs	TANs bind the Fc region of mAbs via Fc receptors (FcRs), triggering ADCC-mediated killing	[48]
	Enhancing Adaptive Immunity	NE	NE enhances activation of CD8^+^ T cells at distant sites.	[45]
		T cell cooperation, iNOS	T cells detect tumor antigens and activate neutrophils to eliminate tumor escape via iNOS.	[49]
		Antigen presentation	Immature neutrophils differentiate into antigen-presenting TANs under IFN-γ and GM-CSF, capturing tumor antigen, migrating to lymph nodes, and activating T cells.	[50,51]
	Immunotherapy Response	Cytokine feedback loop	IL-12 released by Dendritic cells and macrophages stimulates T cells to produce IFN-γ, which activates IRF1 in neutrophils and thus amplifies antitumor activity and feedback to macrophages and T cells.	[52,53]
	NET-Mediated Tumor killing	NETs	NETs trap CTCs via β1-integrin interactions, limiting metastatic spread. NETs also carry cytotoxic proteins including NE and MPO that can damage tumor cells.	[55]
N2	Tumor Initiation	Genetic instability caused by NO, ROS, and Oncogenic miRNAs (miR-23a & miR-155)	Chronic NO/ROS cause DNA damage. Neutrophil-derived vesicles deliver miR-23a and miR-155, inducing DNA double-strand breaks and promoting carcinogenesis.	[56,57,58]
	Tumor Proliferation	NE	NE degrades IRS-1, increasing PI3K-PDGFR interaction thus promoting cell proliferation.	[59]
		PGE_2_	Neutrophil-secreted PGE_2_ promotes RAS-driven proliferation	[60]
		Neutrophil senescence, APOE-TREM2 interaction, SASP, IL-1RA	APOE produced by tumor cells binds to TREM2, activating the downstream DAP12/SYK pathway and promoting neutrophil senescence. These senescent neutrophils adopt the SASP phenotype, secreting pro-inflammatory cytokines as well as IL-1RA, thus promoting tumor proliferation.	[61,62,63]
		NET	NETosis is triggered by tumor-released IL-8, RAGE ligands, and Amyloid β and can promote tumor cell proliferation via the NF-κB signaling pathway.	[64,65,66,67]
	Tumor Angiogenesis	Proangiogenic factors (Bv8/Prok2, VEGF, MMP-9, OSM, FGF2)	Neutrophils release VEGF, Bv8/Prok2, and FGF2; MMP-9 liberates ECM-bound VEGF; OSM activates JAK–STAT to upregulate VEGF in tumors.	[68,69,70,71,72]
	Tumor Metastasis	EMT Inducers (IL-17, TGF-β, and NE)	Neutrophil-released IL-17, TGF-β, and NE induce EMT, reduce adhesion, and enhance tumor invasion.	[73,74,75]
		NETs	NETs remodel ECM via NE and MMP-9, awaken dormant tumor cells, and trap CTCs.	[65,76]
		Adhesion and Energy Transfer	Neutrophils bind to CTCs via β2 integrin–ICAM-1, protect from shear and immune attack, and transfer lipids to fuel metastasis.	[77,78,79]
	Immune Suppression	Nutrient Depletion, cytokines, PD-L1	Neutrophils consume glucose, produce lactic acid and PGE2, express PD-L1, and secrete IL-10/IL-1β, thereby suppressing T cells and promoting macrophages polarization.	[15,41,55,80,81,82,83]

### 2.2. Pro-Tumor Functions of TANs

N2-polarized TANs support tumor progression through multiple mechanisms, including promoting tumor initiation and proliferation, enhancing angiogenesis and metastasis, suppressing anti-tumor immunity, and facilitating therapy resistance (Figure 1; Table 1) [41].

TANs promote tumor initiation through the induction of genetic instability and a pro-inflammatory environment that favors tumor development. Particularly, TANs produce high levels of NO during chronic inflammation and induce ROS such as H_2_O_2_, which cause DNA damage and oxidative stress, resulting in genetic instability and promoting cancer formation in colorectal and intestinal tumor models [56,57]. Additionally, neutrophil-derived extracellular vesicles carrying oncogenic miRNAs such as miR-23a and miR-155 are delivered to epithelial cells, where they induce double-strand DNA breaks and contribute to tumor initiation [58].

TANs further contribute to tumor proliferation through the secretion of PGE2 in a zebrafish RAS-driven tumor model [60] and through NE-mediated degradation of insulin receptor substrate-1 (IRS-1), which enhances interactions between phosphatidylinositol 3-kinase (PI3K) and the potent mitogen platelet-derived growth factor receptor (PDGFR), promoting tumor cell proliferation [59]. In prostate cancer, tumor-derived apolipoprotein E (APOE) binds to triggering receptor expressed on myeloid cells 2 (TREM2) on neutrophils, activating DAP12/SYK signaling. This pathway promotes neutrophil senescence characterized by the senescence-associated secretory phenotype (SASP), which suppresses NK cells and CD8+ T cells and drives chronic inflammation and tumor proliferation [61,62,63]. In a murine and human breast cancer model, senescent TANs promote therapy resistance by secreting SASP-associated exosomes containing piRNA-17560, regulated through the STAT3 signaling pathway [84]. TANs also release interleukin-1 receptor antagonists (IL-1RA) to shield tumor cells from entering senescence, sustaining tumor proliferation [62]. Moreover, tumor-derived IL-8, RAGE ligands, and amyloid β induce NET formation via ROS and inflammatory signaling, promoting tumor proliferation through the activation of nuclear factor kappa-light-chain-enhancer of activated B cells (NF-κB) [64,65,85].

In addition to supporting tumor growth, TANs contribute significantly to tumor angiogenesis by releasing proangiogenic factors such as VEGF, prokineticin-2 (Bv8/Prok2), and fibroblast growth factor 2 (FGF2) [68,69,70]. TANs also secrete MMP-9, which liberates VEGF from the (extracellular matrix) ECM, boosting angiogenesis [71]. In breast cancer, TAN-derived oncostatin M (OSM) activates the JAK-STAT pathway in tumor cells, promoting VEGF expression and angiogenesis [72].

For tumor metastasis, TANs facilitate epithelial–mesenchymal transition (EMT) via IL-17, TGF-β, and NE [73,74,75], decreasing cell adhesion and enhancing tumor cell motility. In breast, pancreatic, and melanoma cancer, chronic inflammation and cancer-associated fibroblasts (CAFs) can trigger NET formation. The NET-associated enzymes NE and MMP-9 remodel laminin, reactivating dormant tumor cells and promoting metastasis [65,66,76]. TANs also act as adhesion partners and escort CTCs to distant sites by forming β2 integrin–ICAM-1 bonds that protect CTCs from shear stress and immune clearance [77,78]. Additionally, TANs accumulate lipids due to suppressed activity of adipose triglyceride lipase (ATGL) in neutrophils, which are later delivered to metastatic tumor cells, supporting energy metabolism and outgrowth in the lung niche [79].

Finally, TANs mediate immune evasion by depleting nutrients like glucose [80], releasing immunosuppressive metabolites such as lactic acid [81] and PGE2 [82], upregulating PD-L1 in response to tumor-derived signals such as TNF-α, GM-CSF, high mobility group box 1 (HMGB1), IL-6, and CCL20 [86,87,88,89,90,91,92], and inducing immunosuppressive cytokines including IL-10 and interleukin-1 beta (IL-1β) [15,63,83].

## 3. Neutrophils in Chemotherapy

**Figure 2:**
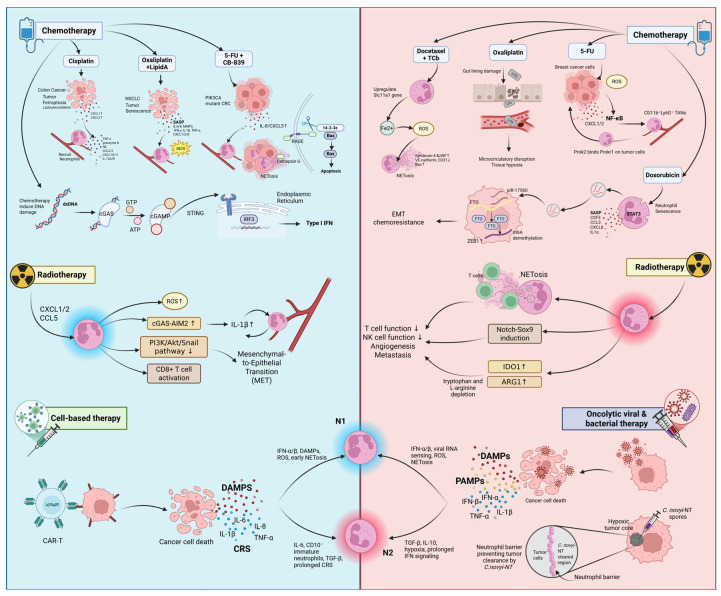
Therapeutic Modulation of Neutrophils in Cancer. Chemotherapy, radiotherapy, cell-based therapy, and oncolytic viral and bacterial therapies can recruit and modulate neutrophil phenotype within TME. Depending on the treatment context and local signaling cues, neutrophils polarize into either antitumor N1 or protumor N2 phenotypes.

Chemotherapy remains a foundational strategy in cancer treatment, primarily functioning by disrupting DNA replication, mitosis, and metabolic pathways essential for tumor cell survival and proliferation [93,94]. However, accumulating evidence suggests that its impact extends beyond direct tumor cytotoxicity. Many chemotherapeutic agents inflict collateral damage on surrounding stromal and immune cells within the TME, resulting in tissue injury and initiating a form of non-infectious, therapy-induced inflammation known as sterile inflammation [95]. This inflammatory response is characterized by the release of damage-associated molecular patterns (DAMPs) from dying or stressed cells, including molecules like HMGB1, adenosine triphosphate (ATP), and calreticulin, alongside the production of pro-inflammatory cytokines such as IL-1β and TNF-α, and chemokines including CXCL1, CXCL2, and CXCL8/IL-8 [95,96,97,98,99]. These inflammatory mediators serve as chemoattractants that recruit innate immune cells, particularly neutrophils. Although traditionally regarded as passive bystanders in cancer therapy, neutrophils are now recognized as dynamic responders to chemotherapy-induced inflammation [100]. Within TME, TANs undergo context-dependent phenotypic programming shaped by cytokine gradients, tumor-derived signals, or the specific chemotherapeutic agents administered. This plasticity enables neutrophils to adopt a functional spectrum ranging from anti-tumorigenic N1-like to pro-tumorigenic N2-like phenotypes (Figure 2; Table 2) [23,32,101].

**Table 2:** Chemotherapy-induced mechanisms of neutrophil polarization in the TME.

Chemo Agent/ Effector	Tumor Model (Species)	Polarization Mechanism	Phenotype	References
Oxaliplatin + Lipid A (OM-174)	Colorectal tumor; PROb (Rat), CT26 (mouse)	Oxaliplatin induces SASP and chemokines (CXCL1/2/8). Lipid A, a TLR4 agonist, promotes iNOS expression and N1 polarization, leading to over 95% tumor regression.	N1	[102,103]
Cisplatin	NSCLC (A549, human)	Cisplatin-induced ferroptosis in tumor cells triggers the release of CXCL1/2 and DAMPs, recruiting neutrophils and polarizing TANs to N1 characterized by the upregulation of TNF-α, granzyme B, and NE. N1 TANs enhance T cell infiltration, CD4^+^ T cell differentiation, and CD8^+^ T cell activation and migration.	N1	[45,104,105,106]
CB-839 + 5-FU/Capecitabine	PIK3CA-mutant CRC (mouse, human clinical phase II trial)	CB-839 and 5-FU/Capecitabine (oral form of 5-FU) combination treatment upregulate IL-8/CXCL5 (human/mice), leading to neutrophil recruitment. This increases ROS and induces NET formation, releasing CTSG that promotes tumor apoptosis via BAX activation.	N1	[107]
DNA damage (cGAS-STING)	Murine tumors, melanoma patients (human)	Chemotherapy-induced DNA damage activates the cGAS-STING pathway and IFN-β signaling, promoting N1 polarization and enhancing cytotoxicity in tumors.	N1	[26,41,108,109]
5-FU	4T1 (mouse, TNBC lung metastasis)	5-FU induces ROS and activates NF-κB, upregulating CXCL1/2 thereby recruiting TANs that express *Prok2*. Promotes angiogenesis and metastasis.	N2	[110]
Docetaxel + Carboplatin (TCb)	Human and mouse breast cancer	Docetaxel and TCb combination therapy upregulates the *Slc11a1* gene in neutrophils, releases Fe^2+^ and ROS, promoting NET formation, which damages endothelium and supports metastasis.	N2	[111]
Doxorubucin	Breast cancer MCF-7, MDA-MB-231 (human); xenograft (mouse)	Doxorubicin induces neutrophil senescence and exosome release via STAT3. Exosomal piR-17560 stabilized FTO and upregulated ZEB1 in tumor cells, promoting EMT and chemoresistance.	N2	[84]
G-CSF (post-chemo)	4T1 (mouse), human lung metastasis	G-CSF restores neutrophils but primes them for NET release and N2 polarization. This promotes metastasis in both human and mouse models.	N2	[112,113,114,115,116]

### 3.1. Chemotherapy-Induced Polarization Toward Antitumor N1 Neutrophils

Several chemotherapeutic agents have been reported to promote N1-like polarization. For instance, in murine colorectal tumor models (rat PROb and mouse CT26), oxaliplatin induces tumor cell senescence characterized by the SASP. This pro-inflammatory state involves elevated mRNA expression of IL-6, IL-8, and MMP-3; increased protein levels of IFN-γ, IL-1β, and TNF-α; and upregulation of neutrophil-attracting chemokines such as CXCL1, CXCL2, and IL-8/CXCL8. These chemokines facilitate neutrophil recruitment into the TME. Upon administration of lipid A (OM-174), a Toll-like receptor 4 (TLR4) agonist, TANs are reprogrammed toward an N1-like phenotype, marked by iNOS activation [102]. These N1 neutrophils enhance local cytotoxic activity, and the combination therapy leads to complete tumor regression in over 95% of treated animals [103].

Similarly, in non-small cell lung cancer (NSCLC) models, cisplatin triggers ferroptosis in tumor cells (A549 human lung cancer cells), as evidenced by lipid peroxidation. This process induces the upregulation of chemokines CXCL1 and CXCL2, which recruit TANs into the TME. Cisplatin-triggered ferroptotic tumor cells reprogram TANs toward an N1-like phenotype, characterized by the upregulation of cytotoxic effectors such as TNF-α, granzyme B, and NE, as well as chemokines and cytokines including CCL2, CCL3, CXCL9, CXCL10, CXCL11, IL-12A, and IL-12B. These N1 TANs exert antitumor effects not only through direct tumor cytotoxicity but also by enhancing immune responses. Specifically, N1 TANs promote CD8^+^ T cell activation and migration through upregulation of T cell–recruiting chemokines (CXCL9/10/11) and facilitate Th1 differentiation of CD4^+^ T cells via IL-12A and IL-12B signaling. Co-culture experiments reveal that CD4^+^ T cells exposed to these TANs exhibit elevated mRNA expression of *IFNA2, IFNB, IFNG, TNF-β, IL-2*, and *CCR5*, along with increased IFN-γ secretion, collectively supporting a robust Th1-skewed immune response [45,104,105,106].

Furthermore, the combination of CB-839, a glutaminase inhibitor, and 5-fluorouracil (5-FU)—or its oral prodrug capecitabine used in a Phase II clinical trial—has been shown to suppress the growth of PIK3CA-mutant colorectal cancers (CRCs) in both murine models and metastatic CRC patients. This chemotherapy regimen induces an N1-like neutrophil response by upregulating IL-8 (human) or CXCL5 (mouse), thereby promoting neutrophil recruitment into the TME. The drug combination increases ROS in neutrophils, triggering NET formation (NETosis) and the release of cathepsin G (CTSG), which enters tumor cells via RAGE, cleaves 14-3-3ε, and activates BAX-dependent mitochondrial apoptosis [117].

Moreover, chemotherapy-induced DNA damage can activate the cyclic GMP-AMP synthase–stimulator of interferon genes (cGAS–STING) pathway by generating cytosolic double-stranded DNA fragments, which trigger a signaling cascade through TANK-binding kinase 1 (TBK1) and IRF3 that results in the transcription and secretion of IFN-β [108]. Type I IFNs, particularly IFN-β, promote neutrophil polarization toward an N1 phenotype, enhancing their antitumor activity in both murine models and human patients [41,109]. In mice, IFN-β deficiency results in N2-skewed TANs and accelerated tumor growth. In melanoma patients treated with type I IFN therapy, neutrophils exhibit increased N1 markers, reduced chemokine receptor expression, and enhanced cytotoxic features, supporting the conserved role of IFN-β in driving N1 polarization across species [26].

### 3.2. Chemotherapy-Induced Polarization Toward Antitumor N2 Neutrophils

On the other hand, some chemotherapeutic agents can paradoxically promote N2-like polarization. In a mouse triple-negative breast cancer (4T1) lung metastasis model, 5-FU elevated ROS in tumor cells, activating NF-κB signaling and upregulating CXCL1 and CXCL2 to recruit neutrophils. These N2-like TANs expressed Prok2, which binds Prokr1 on 4T1 tumor cells, supporting metastatic outgrowth and angiogenesis [110].

Additionally, in a neoadjuvant breast cancer model involving both human and mouse systems, combination therapy with docetaxel and carboplatin (TCb) was shown to induce NET formation through upregulation of the *Slc11a1* gene in neutrophils. *Slc11a1* encodes NRAMP1, a transporter responsible for increasing ferrous iron (Fe^2+^) influx into neutrophils, which in turn enhances intracellular ROS production and promotes NET release. These chemotherapy-induced NETs contribute to vascular endothelial injury, as evidenced by elevated levels of endothelial damage markers such as Syndecan-4 and von Willebrand Factor (vWF), decreased expression of VE-cadherin and CD31, and increased expression of the pro-apoptotic marker Bax in human umbilical vein endothelial cells (HUVECs). Together, these findings suggest a polarization toward an N2-like neutrophil phenotype, characterized by pro-metastatic and tissue-damaging activities [111].

Similarly, oxaliplatin was shown to robustly induce NET formation by activating neutrophils through elevated ROS, MPO, and Peptidyl Arginine Deiminase 4 (PAD4) signaling pathways. This process is initiated by oxaliplatin-induced damage to the gut lining, which allows bacterial lipopolysaccharide (LPS) to leak into the bloodstream, further stimulating neutrophil activation. The resulting NETosis contributes to microcirculatory disruption and tissue hypoxia [118].

Recent studies have identified senescent neutrophils as key contributors to chemoresistance in breast cancer. In response to doxorubicin, neutrophils undergo senescence—evidenced by the upregulation of p16 ^INK4A^ and elevated expression of SASP factors such as colony stimulating factors 3 (CSF3), CCL3, CXCL8, and IL-1α. Senescent neutrophils exhibit enhanced exosome secretion. Activated STAT3 signaling in these neutrophils drives the packaging of PIWI-interacting RNA piR-17560 into exosomes. Once transferred to breast cancer cells, piR-17560 stabilizes FTO mRNA, increasing FTO protein levels and enabling m6A demethylation of ZEB1 mRNA, which prevents its degradation by the m6A reader YTHDF2. This results in increased ZEB1 expression, which promotes EMT, characterized by downregulation of E-cadherin and upregulation of Vimentin, ultimately enhancing tumor cell invasiveness and resistance to docetaxel [84].

Beyond its direct cytotoxic effects, chemotherapy can also influence neutrophil behavior through downstream consequences such as treatment-induced neutropenia, which is defined as a significant reduction in circulating neutrophils due to bone marrow suppression [119]. This condition increases susceptibility to opportunistic infections and often necessitates dose delays or reductions [120]. To counteract this, G-CSF is commonly administered to stimulate neutrophil production and restore immune competence [112,113,114]. However, emerging evidence suggests that G-CSF not only increases neutrophil counts but also promotes their polarization toward an N2-like, pro-tumorigenic phenotype characterized by enhanced NET formation. In the 4T1 murine breast cancer model, tumor-derived G-CSF was shown to prime neutrophils for NET release, which could be reversed by anti-G-CSF treatment. Moreover, neutrophils from healthy mice also became NET-prone upon treatment with recombinant G-CSF, indicating a direct role for G-CSF in driving this phenotype [115]. A subsequent study extended these findings to humans, reporting elevated NET levels in lung metastases compared to primary breast tumors and confirming that recombinant G-CSF could induce NET formation in human neutrophils ex vivo, further supporting its role in promoting pro-metastatic neutrophil behavior [116].

### 3.3. Therapeutic Strategies Targeting N2 Neutrophils to Enhance Chemotherapy Response

The recognition of neutrophils’ pro-tumorigenic roles in chemotherapy has spurred the development of several therapeutic strategies aimed at reprogramming or inhibiting N2-like phenotypes. One effective approach targets NET formation. PAD4 inhibitors, such as Cl-amidine and GSK484, inhibit chromatin citrullination, thereby reducing NET production, metastatic potential, and enhancing chemotherapy responsiveness in preclinical models [116,121]. Likewise, Deoxyribonuclease 1 (DNase1) has been shown to safely degrade NETs across various murine models, including breast cancer, pulmonary inflammation, and autoimmune diseases, with no significant toxicity reported in non-cancer clinical use [122]. Another strategy is the use of combination chemo–immunotherapy regimens, which have shown potential to reduce N2-associated TANs while enhancing antitumor immune responses. For example, in colorectal carcinoma models, combining oxaliplatin with ATR inhibition and anti-PD-1 therapy significantly decreased neutrophil infiltration and facilitated the expansion of stem-like, IFN-γ–producing CD8^+^ T cells, resulting in complete tumor regression [123]. Together, these findings reinforce the therapeutic promise of targeting neutrophil plasticity to enhance chemotherapy efficacy and limit tumor progression.

## 4. Neutrophils in Radiotherapy (RT)

RT is another fundamental treatment modality in oncology that employs ionizing radiation, such as high-energy X-rays or subatomic particles, to destroy cancer cells. This is primarily achieved by inducing double-strand DNA breaks, thereby disrupting the cell cycle and leading to mitotic catastrophe, a form of cell death caused by irreparable division failure [124,125,126]. Although normal tissues are also exposed, their greater capacity for DNA repair allows selective survival when treatment is fractionated over time [127]. Depending on the clinical scenario, RT may be applied externally (external beam radiation) or internally (brachytherapy), either as a standalone therapy or in combination with surgery, chemotherapy, or immunotherapy [128,129]. RT triggers a robust cytokine and chemokine cascade within the TME that plays a central role in neutrophil recruitment. Across multiple preclinical tumor models, RT has been shown to increase the expression of cytokines such as IL-1β, GM-CSF (CSF2), and G-CSF (CSF3), along with chemokines including CXCL1, CXCL2, CXCL5, CCL2, and CCL5 [130]. IL-1β can further amplify this response by upregulating CXCL chemokines, thereby enhancing neutrophil-attracting signals. In parallel, RT elevates the expression of chemokine receptors CXCR2 and CXCR4 on neutrophils, which bind to CXCL1 and CXCL2, promoting their directed migration into the irradiated TME [130,131,132,133,134,135]. Beyond the local tumor site, RT can also alter systemic cytokine levels, supporting neutrophil mobilization and expansion in the circulation. Clinical observations align with these findings; in patients with pancreatic cancer, elevated levels of CCL2, CCL4, TGF-β, and VEGF were detected in the blood after RT, suggesting that radiation not only reshapes the TME but also exerts systemic immunomodulatory effects that may influence neutrophil behavior [136] (Figure 2; Table 3).

**Table 3:** Radiotherapy-induced mechanisms of neutrophil polarization in the TME.

Tumor Model (Species)	Polarization Mechanism	Phenotype	References
LLC model (Mouse)	RT-induced DNA damage increases CXCL1, CXCL2, and CCL5 expression, recruiting ROS-producing neutrophils. G-CSF also enhances neutrophil recruitment. ROS generation in combination with RT suppresses PI3K/Akt/Snail signaling, inhibiting EMT and promoting MET.	N1	[137]
RM-9 prostate, EG7 thymoma, 4T1 breast (Mouse)	RT rapidly recruits CD11b^+^Gr-1 ^high+^ neutrophils, which produce ROS that triggers tumor apoptosis and initiate sterile inflammation, enhancing CTL activation.	N1	[75]
In vitro (Human or Mouse); thymoma, breast, prostate, pancreatic (Mouse)	Higher RT doses enhance neutrophil ROS production, contributing to tumor regression; ROS inhibition reduces the antitumor effect.	N1	[138,139,140]
MC38 colorectal and RM-9 prostate (Mouse)	RT activates cGAS and AIM2 pathways, increasing IL-1β expression, which in turn elevates CXCL chemokines and drives neutrophil infiltration.	N1	[131]
Lung tissue pre-metastatic niche with breast cancer cells (Mouse)	RT recruits activated neutrophils to irradiated lung tissue, which promotes Notch–Sox9 signaling in infiltrating cancer cells, inducing stem-like, pro-metastatic traits.	N2	[141]
Cervical cancer (Human)	High peripheral neutrophil counts during treatment correlate with poor local control and survival, suggesting a protumorigenic role for TANs in clinical settings.	N2	[142]
Bladder cancer (Mouse and Human)	RT induces robust NET formation that physically blocks CD8^+^ T cells from accessing tumor cells and impairs cytotoxicity, contributing to immune evasion and treatment resistance.	N2	[143]
In vitro colon carcinoma spheroids (Human)	Low RT dose (e.g., 0.25 Gy) stimulates NET formation that restricts immune cell-mediated tumor killing.	N2	[144]
Prostate and pancreatic cancer (Mouse); Rectal cancer (Human)	RT increases expression of IDO1 and ARG1 in TANs, which deplete tryptophan and L-arginine, suppressing CD8^+^ T cells and NK cell functions and weakening antitumor immunity.	N2	[136,145,146]

### 4.1. RT-Induced Polarization Toward Antitumor N1 Neutrophils

RT has been shown to actively reprogram TANs toward an antitumor, N1-like phenotype through multiple converging mechanisms involving DNA damage response, inflammatory signaling, and ROS production [130,147]. In mice bearing Lewis lung carcinoma (LLC), hypofractionated RT (8 Gy × 3) induced persistent DNA double-strand breaks, upregulated chemokines such as CXCL1, CXCL2, and CCL5, and recruited newly infiltrating neutrophils into the TME [137]. These RT-recruited TANs generated elevated levels of ROS, which suppressed the PI3K/Akt/Snail signaling cascade, thereby inhibiting EMT and promoting mesenchymal–epithelial transition (MET). This effect sensitized tumors to radiation and constrained progression. Co-administration of G-CSF further amplified this N1 phenotype by enhancing neutrophil recruitment, ROS generation, and MET induction [137]. Similarly, in syngeneic mouse models including RM-9 prostate cancer, EG7 thymoma, and 4T1 breast cancer, a single high dose of RT (15 Gy) caused rapid infiltration of CD11b^+^Gr-1 ^high+^ neutrophils within 24 h. These TANs generated high levels of ROS, induced tumor cell apoptosis, and initiated sterile inflammation that facilitated activation of tumor-specific CTLs. G-CSF synergized with RT to further strengthen these effects [138]. In syngeneic murine colorectal (MC38) and prostate (RM-9) tumor models, RT activated the cGAS and AIM2 DNA-sensing pathways, leading to IL-1β upregulation and CXCL chemokine-mediated neutrophil recruitment [131]. Additionally, murine models of thymoma, breast, prostate, and pancreatic cancers demonstrated that RT significantly increased neutrophil-derived ROS within tumors, contributing to tumor regression in a dose-dependent manner [138,139,140]. Notably, in vitro studies revealed that single RT doses of 6, 12, or 18 Gy enhanced neutrophil ROS release, while fractionated RT reduced ROS production compared to unirradiated controls [148].

### 4.2. RT-Induced Polarization Toward Antitumor N2 Neutrophils

In contrast to its capacity for promoting N1 phenotypes, RT can also skew neutrophils toward a tumor-promoting, N2-like state, facilitating metastasis and radioresistance through diverse mechanisms [149]. In a thoracic RT model, Nolan et al. demonstrated that irradiation of healthy lung tissue led to the recruitment of activated neutrophils, which induced Notch–Sox9 signaling in infiltrating breast cancer cells, promoting stem-like and metastatic traits. Inhibiting neutrophil degranulation reversed this effect, implicating TAN-derived factors in the formation of pro-metastatic niches [141]. Clinically, elevated neutrophil levels have been linked to poor outcomes following RT. In cervical cancer patients treated with chemoradiotherapy, high peripheral neutrophil counts during treatment were associated with worse local control and survival. Using a genetically engineered mouse model of autochthonous sarcoma, Wisdom et al. showed that both genetic and antibody-mediated neutrophil depletion improved tumor sensitivity to image-guided focal irradiation, with corresponding downregulation of Mitogen-Activated Protein Kinase (MAPK) pathway activity. These findings support a functional role for neutrophils in promoting tumor resistance to radiation [142]. At the mechanistic level, NETs have emerged as key mediators of N2-like functions in irradiated tumors. Shinde-Jadhav et al. reported that RT induces NET formation in murine bladder tumors, particularly at high or fractionated doses. These fibrous traps impede CD8^+^ T cell infiltration and cytotoxic activity. Disruption of NETs via PAD4 knockout, DNase I, or NE inhibition significantly improved therapeutic efficacy and survival [143]. NETosis was also observed in vitro at RT doses as low as 0.25 Gy and increased with dose escalation [144]. In vivo, colon and bladder tumors exhibited NET-mediated immune exclusion, while bladder cancer patients with residual disease post-RT showed elevated NET levels and worse outcomes [143,144]. In parallel, RT upregulates immunosuppressive enzymes such as indoleamine 2,3-dioxygenase 1 (IDO1) and ARG1 in TANs, which deplete tryptophan and L-arginine—amino acids essential for CD8^+^ T and NK cell function. This phenomenon was demonstrated in murine prostate and pancreatic tumor models [136,145] and in rectal cancer patients where circulating neutrophils exhibited high ARG1 expression [146]. IDO1 also expands regulatory T cells, suppresses NK cell activity, and promotes angiogenesis [150,151,152,153]. Combining ARG1 inhibition with RT reduced TAN infiltration, restored CD8^+^ T cell presence, and delayed tumor growth [145,153].

### 4.3. Dose-Dependent Effects of RT on Neutrophil Function

The immunological effects of RT on neutrophils are highly dose- and context-dependent. In radiosensitive tumor models such as EG7-bearing C57BL/6 mice, a low dose of 1.3 Gy was sufficient to elicit antitumor responses via infiltration of CD11b^+^Gr-1^high^+^ neutrophils and suppression of tumor viability [138,154]. In contrast, more resistant models like 4T1 breast cancer required a higher dose (15 Gy) to achieve comparable ROS-mediated antitumor effects [138]. However, high-dose regimens (≥10 Gy) can also trigger NETosis, which may promote tumor progression. In a bladder cancer model, 10 Gy or 2 × 5 Gy radiation significantly increased NET formation, whereas 2 Gy failed to do so. These NETs formed extracellular barriers that physically excluded CD8^+^ T cells, thereby limiting T cell infiltration and cytotoxic activity and contributing to radioresistance [143]. Meanwhile, in the Lewis lung carcinoma (LLC) model, fractionated 8 Gy × 3 RT induced CXCL1/2/5 chemokines and robust ROS production, promoting mesenchymal-to-epithelial transition (MET) and enhancing tumor radiosensitivity [137]. Collectively, these findings highlight how both RT dose intensity and tumor type influence neutrophil polarization and function, with critical implications for therapeutic efficacy [154].

## 5. Neutrophils in Cell-Based Therapies

Cell-based cancer immunotherapies have introduced a new method in oncology by employing immune cells to selectively target and eliminate tumor cells [28]. These strategies include well-established modalities such as adoptive T cell transfer, chimeric antigen receptor (CAR) T-cell therapy, and newer approaches involving NK cells and macrophage-based therapies [155,156,157,158]. While these treatments primarily rely on direct immune-mediated tumor cell killing, their therapeutic outcomes are significantly influenced by the surrounding TME [159,160]. Among its many components, neutrophils, particularly TANs, have emerged as important regulators that can either support or impair the effectiveness of these immunotherapies (Figure 2) [52,161].

In CAR-T cell therapy, neutrophils have been implicated in both therapeutic response and treatment-related toxicities [162,163,164,165]. Several studies have reported that activated neutrophils, and particularly NETosis, are involved in the pathogenesis of cytokine release syndrome (CRS), an acute systemic inflammatory response triggered by activated CAR-T cells [162,163,164]. For instance, longitudinal plasma proteomics from patients treated with CAR-T cells revealed a temporal association between markers of neutrophil activation, NET formation, and the onset of CRS [165]. Clinical data further show that absolute neutrophil count (ANC) at baseline and during therapy correlates with CAR-T cell expansion kinetics and the likelihood of CRS, suggesting that neutrophils may serve as early biomarkers of treatment dynamics [162].

Moreover, neutrophil phenotypes appear to shape clinical outcomes. One study found that elevated frequencies of CD10^−^ immature neutrophils were associated with poor responses and inferior survival in patients receiving CD19-directed CAR-T therapy for B-cell acute lymphoblastic leukemia [166]. These immature neutrophils may contribute to an immunosuppressive environment that impairs CAR-T function. Notably, a recent single-cell study of ciltacabtagene autoleucel–treated myeloma patients demonstrated that CRS is accompanied by a wave of neutrophil activation that precedes clonal CAR-T cell re-expansion, suggesting a functional link between innate and adaptive cellular dynamics during therapy [165].

Beyond acting as modulators of other immune cell therapies, neutrophils themselves are now being explored as direct therapeutic agents [27]. Recent advances in stem cell engineering have enabled the generation of CAR-neutrophils derived from human pluripotent stem cells (hPSCs) [167,168,169]. These engineered neutrophils exhibit potent antitumor activity against solid tumors in vivo, including glioblastoma and prostate cancer models [167,169,170]. Notably, CAR-neutrophils retain their natural tumor-homing capacity while being redirected to specifically recognize and kill tumor cells through CAR-mediated signaling. In glioblastoma models, CAR-neutrophils have also been used as delivery vehicles for TME–responsive nanodrugs, providing a combinatorial platform for chemoimmunotherapy [170].

## 6. Neutrophils in Oncolytic Viral (OVT) and Bacterial Therapies

OVT represents another promising class of cancer treatment that utilizes replication-competent viruses to selectively infect, lyse tumor cells, and activate systemic antitumor immunity [171]. Several oncolytic viruses, including modified herpes simplex virus (HSV), adenovirus, vaccinia virus, have been developed, with some already approved for clinical use [172,173,174]. In addition to direct tumor lysis, OVT stimulates immunogenic cell death, releases tumor antigens, and recruits immune effector cells, thereby facilitating crosstalk between innate and adaptive immune responses [175,176]. Neutrophils have recently found a critical yet underappreciated role in shaping OVT outcomes (Figure 2) [177,178].

On the one hand, neutrophils can restrict OVT effectiveness by clearing therapeutic viruses prematurely. These effects are mediated through phagocytosis, inflammatory cytokine release, and NET formation. For example, in glioma models treated with oncolytic HSV, tumor-derived G-CSF triggered neutrophil-driven NETosis, which limited viral propagation and impaired tumor control. Inhibiting this pathway improved therapeutic outcomes. [179]. Similarly, transient blockade of neutrophil activity improved systemic delivery of oncolytic vaccinia virus by reducing early viral clearance [180]. Conversely, when properly activated, neutrophils can enhance OVT by mediating direct cytotoxicity and shaping downstream immunity. In a pulmonary melanoma model, oncolytic Orf virus (ORFV)–induced neutrophils contributed to tumor regression and supported viral amplification [181]. Neutrophils have also been shown to secrete TNF-α which facilitates antigen presentation and T cell priming during OVT, thereby linking innate and adaptive immunity [181].

**Figure 3:**
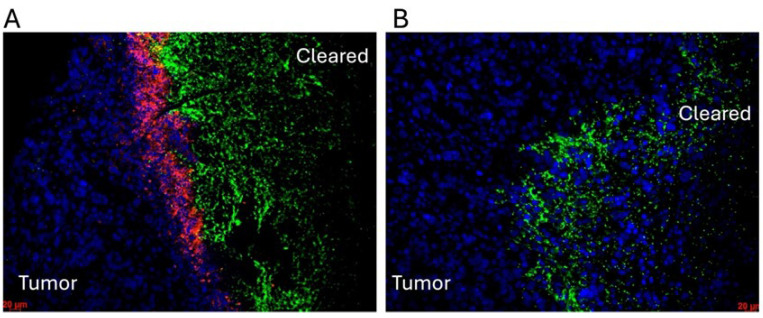
Tumor clearance by *C. novyi*-NT is inhibited by neutrophil barriers in a subcutaneous GL261 tumor model. (**A**) Mice with flank-implanted GL261 tumors received intratumoral injections of *C. novyi*-NT spores. After 12 h, tumors were harvested, fixed, and stained with anti-Ly6G (1A8, red) and anti-*C. novyi* (green) antibodies. The region labeled “Cleared” indicates complete tumor eradication. A neutrophil barrier was observed between the germinating *C. novyi*-NT and the surrounding tumor tissue. Scale bar: 20 µm. (**B**) Mice with flank-implanted GL261 tumors were administered anti-Ly6G antibody intraperitoneally (IP) 24 h before intratumoral injection of *C. novyi*-NT spores. Tumors were collected and stained as described in panel A. The formation of a neutrophil barrier was not detected. Scale bar: 20 µm.

In addition to oncolytic viruses, the use of *Clostridium novyi*-NT (*C. novyi-NT*) has emerged as a promising modality to treat hypoxic and treatment-resistant tumors (Figure 2). *C. novyi*-NT is an obligate anaerobe capable of selectively germinating within hypoxic tumor cores and inducing localized tumor necrosis [182,183]. Over the past decade, our group and others have demonstrated the potent antitumor effects of *C. novyi*-NT across multiple models, including orthotopic glioblastomas [184,185,186,187,188]. We also highlighted *C. novyi*-NT as a paradigm for hypoxia-targeting bacterial cancer therapy [182]. A critical insight from our work is that host neutrophil responses significantly influence the therapeutic outcomes of oncolytic bacterial therapy. This concept was directly demonstrated in our studies. Immunofluorescence analysis revealed that Ly6G^+^ neutrophils form a localized barrier around germinating *C. novyi*-NT spores following intratumoral injection, physically separating the bacteria from surrounding tumor tissue (Figure 3). Notably, neutrophil depletion markedly enhanced bacterial spread and improved tumor clearance in vivo, confirming that neutrophils can act as early barriers to effective bacterial-mediated oncolysis [189].

These findings mirror the challenges faced in OVT, emphasizing a broader principle across microbial therapies: while neutrophils are essential for containing infection and regulating inflammation, they may also hinder microbial propagation within tumors. Thus, modulating neutrophil timing and function represents a promising strategy to optimize the balance between microbial replication and anti-tumor immune activation.

## 7. Conclusions

The past decade has revealed neutrophils to be more than supporting players of innate immunity; they are dynamic, context-dependent regulators of cancer therapy outcomes. Across multiple modalities such as chemotherapy, radiotherapy, cell-based immunotherapy, and oncolytic virotherapy, neutrophils exhibit remarkable functional plasticity. They may adopt either anti-tumor or pro-tumor phenotypes depending on tumor microenvironmental cues. N1-like neutrophils can exert cytotoxicity and promote immune activation, whereas N2-polarized subsets often contribute to tumor progression through mechanisms such as immunosuppression, angiogenesis promotion, and remodeling of the ECM [23,190].

The complexity of neutrophil behavior becomes especially apparent in the context of therapy-induced reprogramming. For instance, type I interferons released following radiotherapy have been shown to promote a tumoricidal N1 phenotype [149,191], whereas cytokines such as IL-6 and G-CSF, which are commonly upregulated in treatment-induced inflammation, tend to reinforce immunosuppressive behaviors and NET formation [85]. These observations emphasize the paradoxical nature of neutrophils: they can either enhance or hinder therapeutic efficacy depending on the timing, location, and context of the treatment. Notably, neutrophil-related effectors, such as ROS, NO, NE, and NETs, along with broader tumor-associated mechanisms such as SASP, act as double-edged swords in cancer. On one hand, ROS can directly kill tumor cells through oxidative bursts and induce apoptosis via TRPM2-mediated calcium influx; on the other, excessive ROS in the TME suppress T cell proliferation and enhance T cell apoptosis. NE similarly promotes tumor cell apoptosis via death receptor cleavage, yet also enhances tumor growth through EGFR signaling and angiogenesis. While NETs can trap and kill CTCs, they more often aid metastasis by facilitating tumor adhesion and extravasation. SASP, which may trigger immune clearance of damaged cells, paradoxically promotes tumor relapse and EMT when persistently secreted after therapy. These examples underscore the challenge of therapeutic targeting in the TME, where context determines whether these neutrophil-driven factors act as allies or adversaries.

A central conceptual challenge moving forward is reconciling the dual roles of neutrophils and the context-dependent effects of their effector functions. While the N1/N2 framework provides a useful starting point, it is increasingly evident that neutrophils span a continuum of activation states, shaped by a dynamic interplay of cytokines, cellular interactions, and therapeutic cues. The traditional binary classification into N1 and N2 is insufficient to capture this complexity. Integrating high-resolution approaches, such as single-cell transcriptomics and spatial proteomics, will be crucial for identifying discrete neutrophil subsets with distinct therapeutic relevance [192,193]. Moreover, longitudinal profiling during therapy, rather than static snapshots, may reveal how neutrophils transition between states and thereby inform the timing of interventions aimed at modulating their activity [194]. In addition to mechanistic insights, peripheral biomarkers such as the neutrophil-to-lymphocyte ratio (NLR) offer a valuable window into neutrophil dynamics during therapy. Elevated NLR has been linked to poor prognosis across multiple tumor types, including hepatocellular carcinoma, NSCLC, and lymphoma [195,196,197,198]. Although NLR does not directly reflect neutrophil phenotype (e.g., N1 vs. N2), it is increasingly recognized as a practical surrogate for systemic inflammation and immune imbalance. This underscores the clinical relevance of tracking neutrophil-related changes during therapy, and further supports the need for therapeutic strategies that consider neutrophil modulation alongside treatment selection.

Ultimately, neutrophils exemplify a broader theme in cancer immunology: innate immune cells are not static tools but responsive, context-sensitive effectors. Deploying their therapeutic potential will require embracing their complexity, accounting for their microenvironment, and intervening with both precision and restraint. The future of neutrophil-targeted cancer therapy will depend not only on better tools, but also on better questions—ones that recognize their duality, adapt to their variability, and seek to direct their power rather than modulate it. This review adopts a pan-cancer perspective, drawing from studies in both solid and hematologic malignancies to illustrate how diverse cancer therapies influence neutrophil phenotypes and functions.

## Abbreviations

The following abbreviations are used in this manuscript:

TME	tumor microenvironment
TANs	Tumor-associated neutrophils
VEGF	Vascular endothelial growth factor
NE	Neutrophil elastase
IL-6/8/10/12/17	Interleukin 6/8/10/12/17
IL-1β	Interleukin-1 beta
ARG1	Arginase 1
NETs	Neutrophil extracellular traps
NETosis	Neutrophil extracellular trap formation
TNF-α/β	Tumor necrosis factor alpha/beta
ROS	Reactive oxygen species
CTLs	Cytotoxic T lymphocytes
NK cells	Natural killer cells
TRAIL	TNF-Related Apoptosis-Inducing Ligand
TGF-β	Transforming growth factor-β
IFN-β/γ	Interferon beta/gamma
RT	Radiotherapy
OVT	Oncolytic virotherapy
NO	Nitric oxide
iNOS	Inducible nitric oxide synthase
ICAM-1	Intercellular Adhesion Molecule 1
CCL2/3/4/5/20	C-C motif chemokine ligand 2/3/4/5/20
CXCL1/2/5/8/9/10/11	C-X-C motif chemokine ligand 1/2/5/8/9/10/11
GM-CSF	Granulocyte-macrophage colony-stimulating factor
PGE2	Prostaglandin E2
G-CSF	Granulocyte colony-stimulating factor
HA	Hyaluronic acid
CXCR2/4	C-X-C Motif Chemokine Receptor 2/4
S100A8/A9	S100 calcium-binding proteins A8/A9
ADCC	Antibody-dependent cytotoxicity
MMP-3/9	Matrix metalloproteinase-3/9
H_2_O_2_	Hydrogen peroxide
HGF	Ligand hepatocyte growth factor
mAbs	Monoclonal antibodies
IRF1/3	Interferon regulatory factor 1/3
CTCs	Circulating tumor cells
MPO	Myeloperoxidase
PI3K	Phosphatidylinositol 3-kinase
PDGFR	Potent mitogen platelet-derived growth factor receptor
APOE	Apolipoprotein E
TREM2	Triggering receptor expressed on myeloid cells 2
SASP	Senescence-Associated Secretory Phenotype
IL-1RA	Interleukin-1 receptor antagonist
NF-κB	Nuclear factor kappa-light-chain-enhancer of activated B cells
Prok2 (Bv8)	Prokineticin-2
FGF2	Fibroblast growth factor 2
OSM	Oncostatin M
EMT	Epithelial–mesenchymal transition
CAF	Cancer-associated fibroblast
ATGL	Activity of adipose triglyceride lipase
HMGB1	High Mobility Group Box 1
DAMPs	Damage-associated molecular patterns
ATP	Adenosine triphosphate
TLR4	Toll-like receptor 4
NSCLC	Non-small cell lung cancer
CCR5	C-C Motif chemokine receptor 5
5-FU	5-fluorouracil
CRCs	Colorectal cancer
CTSG	Cathepsin G
cGAS	Cyclic GMP-AMP synthase
STING	Stimulator of interferon genes
TBK1	TANK-binding kinase 1
ECM	Extracellular matrix
vWF	von Willebrand Factor
HUVECs	umbilical vein endothelial cells
PAD4	Peptidyl Arginine Deiminase 4
CSF3	Colony stimulating factors 3
BAX	Bcl-2-associated X protein
APC	Antigen presenting cells
*IFNA2/B/G*	Interferon alpha 2/beta/gamma
RAGE	Receptor for Advanced Glycation Endproducts
FTO	Obesity-associated protein
piR	piRNA
STAT3	Signal transducer and activator of transcription 3
TRPM2	Transient receptor potential cation channel, subfamily M, member 2
IRS-1	Insulin receptor substrate-1
DNase1	Deoxyribonuclease 1
FasL	Fas ligand
FcRs	Fc Receptors
Th1	T helper type 1
LLC	Lewis lung carcinoma
MET	Mesenchymal–epithelial transition
AIM2	Absent in Melanoma 2
Sox9	SRY-Box Transcription Factor 9
IDO1	Indoleamine 2,3-dioxygenase 1
MAPK	Mitogen-Activated Protein Kinase
CAR	Chimeric antigen receptor
CRS	Cytokine release syndrome
ANC	Absolute neutrophil count
hPSCs	Human pluripotent stem cells
HSV	Herpes simplex virus
ORFV	Oncolytic Orf virus
*C. novyi*-NT	*Clostridium novyi*-NT
NLR	Neutrophil-to-lymphocyte ratio

## References

[1] Tigner A., Ibrahim S.A., Murray I.V. (2022). Histology, White Blood Cell. StatPearls.

[2] Prinyakupt J., Pluempitiwiriyawej C. (2015). Segmentation of White Blood Cells and Comparison of Cell Morphology by Linear and Naïve Bayes Classifiers. Biomed. Eng. Online.

[3] Beyrau M., Bodkin J.V., Nourshargh S. (2012). Neutrophil Heterogeneity in Health and Disease: A Revitalized Avenue in Inflammation and Immunity. Open Biol..

[4] Ng L.G., Ostuni R., Hidalgo A. (2019). Heterogeneity of Neutrophils. Nat. Rev. Immunol..

[5] Chen S., Zhang Q., Lu L., Xu C., Li J., Zha J., Ma F., Luo H.R., Hsu A.Y. (2022). Heterogeneity of Neutrophils in Cancer: One Size Does Not Fit All. Cancer Biol. Med..

[6] Jensen H.K., Donskov F., Marcussen N., Nordsmark M., Lundbeck F., Von Der Maase H. (2009). Presence of Intratumoral Neutrophils Is an Independent Prognostic Factor in Localized Renal Cell Carcinoma. J. Clin. Oncol..

[7] Lee T.-H., Avraham H., Lee S.-H., Avraham S. (2002). Vascular Endothelial Growth Factor Modulates Neutrophil Transendothelial Migration via Up-Regulation of Interleukin-8 in Human Brain Microvascular Endothelial Cells. J. Biol. Chem..

[8] Jablonska E., Piotrowski L., Jablonski J., Grabowska Z. (2002). VEGF in the Culture of PMN and the Serum in Oral Cavity Cancer Patients. Oral Oncol..

[9] Gong Y., Koh D.-R. (2010). Neutrophils Promote Inflammatory Angiogenesis via Release of Preformed VEGF in an in Vivo Corneal Model. Cell Tissue Res..

[10] Lerman I., Garcia-Hernandez M.d.l.L., Rangel-Moreno J., Chiriboga L., Pan C., Nastiuk K.L., Krolewski J.J., Sen A., Hammes S.R. (2017). Infiltrating Myeloid Cells Exert Protumorigenic Actions via Neutrophil Elastase. Mol. Cancer Res..

[11] Teijeira A., Garasa S., Ochoa M.C., Villalba M., Olivera I., Cirella A., Eguren-Santamaria I., Berraondo P., Schalper K.A., de Andrea C.E. (2021). IL8, Neutrophils, and NETs in a Collusion against Cancer Immunity and Immunotherapy. Clin. Cancer Res..

[12] García-Navas R., Gajate C., Mollinedo F. (2021). Neutrophils Drive Endoplasmic Reticulum Stress-Mediated Apoptosis in Cancer Cells through Arginase-1 Release. Sci. Rep..

[13] Zhao Z., Liu T., Liang Y., Cui W., Li D., Zhang G., Deng Z., Chen M., Sha K., Xiao W. (2022). N2-Polarized Neutrophils Reduce Inflammation in Rosacea by Regulating Vascular Factors and Proliferation of CD4+ T Cells. J. Investig. Dermatol..

[14] Griffin G.K., Newton G., Tarrio M.L., Bu D., Maganto-Garcia E., Azcutia V., Alcaide P., Grabie N., Luscinskas F.W., Croce K.J. (2012). IL-17 and TNF-α Sustain Neutrophil Recruitment during Inflammation through Synergistic Effects on Endothelial Activation. J. Immunol..

[15] Coffelt S.B., Kersten K., Doornebal C.W., Weiden J., Vrijland K., Hau C.-S., Verstegen N.J.M., Ciampricotti M., Hawinkels L.J.A.C., Jonkers J. (2015). IL-17-Producing Γδ T Cells and Neutrophils Conspire to Promote Breast Cancer Metastasis. Nature.

[16] Beauvillain C., Delneste Y., Scotet M., Peres A., Gascan H., Guermonprez P., Barnaba V., Jeannin P. (2007). Neutrophils Efficiently Cross-Prime Naive T Cells in Vivo. Blood.

[17] Eruslanov E.B., Bhojnagarwala P.S., Quatromoni J.G., Stephen T.L., Ranganathan A., Deshpande C., Akimova T., Vachani A., Litzky L., Hancock W.W. (2014). Tumor-Associated Neutrophils Stimulate T Cell Responses in Early-Stage Human Lung Cancer. J. Clin. Investig..

[18] Spörri R., Joller N., Hilbi H., Oxenius A. (2008). A Novel Role for Neutrophils As Critical Activators of NK Cells. J. Immunol..

[19] Ralph S.J., Reynolds M.J. (2023). Intratumoral Pro-Oxidants Promote Cancer Immunotherapy by Recruiting and Reprogramming Neutrophils to Eliminate Tumors. Cancer Immunol. Immunother. CII.

[20] Koga Y., Matsuzaki A., Suminoe A., Hattori H., Hara T. (2004). Neutrophil-Derived TNF-Related Apoptosis-Inducing Ligand (TRAIL): A Novel Mechanism of Antitumor Effect by Neutrophils. Cancer Res..

[21] Kundu M., Greer Y.E., Lobanov A., Ridnour L., Donahue R.N., Ng Y., Ratnayake S., Voeller D., Weltz S., Chen Q. (2024). TRAIL-Induced Cytokine Production via NFKB2 Pathway Promotes Neutrophil Chemotaxis and Immune Suppression in Triple Negative Breast Cancers. bioRxiv.

[22] Powell D.R., Huttenlocher A. (2016). Neutrophils in the Tumor Microenvironment. Trends Immunol..

[23] Fridlender Z.G., Sun J., Kim S., Kapoor V., Cheng G., Ling L., Worthen G.S., Albelda S.M. (2009). Polarization of Tumor-Associated Neutrophil Phenotype by TGF-β: “N1” versus “N2” TAN. Cancer Cell.

[24] Zhang X., Huang X., Zhang X., Lai L., Zhu B., Lin P., Kang Z., Yin D., Tian D., Chen Z. (2025). The miR-941/FOXN4/TGF-β Feedback Loop Induces N2 Polarization of Neutrophils and Enhances Tumor Progression of Lung Adenocarcinoma. Front. Immunol..

[25] Pylaeva E., Lang S., Jablonska J. (2016). The Essential Role of Type I Interferons in Differentiation and Activation of Tumor-Associated Neutrophils. Front. Immunol..

[26] Andzinski L., Kasnitz N., Stahnke S., Wu C.-F., Gereke M., Von Köckritz-Blickwede M., Schilling B., Brandau S., Weiss S., Jablonska J. (2016). Type I IFN s Induce Anti-tumor Polarization of Tumor Associated Neutrophils in Mice and Human. Int. J. Cancer.

[27] Zhang J., Gu J., Wang X., Ji C., Yu D., Wang M., Pan J., Santos H.A., Zhang H., Zhang X. (2024). Engineering and Targeting Neutrophils for Cancer Therapy. Adv. Mater..

[28] Paucek R.D., Baltimore D., Li G. (2019). The Cellular Immunotherapy Revolution: Arming the Immune System for Precision Therapy. Trends Immunol..

[29] Chen X., Chen B., Zhao H. (2025). Role of Neutrophils in Anti-Tumor Activity: Characteristics and Mechanisms of Action. Cancers.

[30] Antuamwine B.B., Bosnjakovic R., Hofmann-Vega F., Wang X., Theodosiou T., Iliopoulos I., Brandau S. (2023). N1 versus N2 and PMN-MDSC: A Critical Appraisal of Current Concepts on Tumor-associated Neutrophils and New Directions for Human Oncology. Immunol. Rev..

[31] Kalafati L., Kourtzelis I., Schulte-Schrepping J., Li X., Hatzioannou A., Grinenko T., Hagag E., Sinha A., Has C., Dietz S. (2020). Innate Immune Training of Granulopoiesis Promotes Anti-Tumor Activity. Cell.

[32] Chen Q., Yin H., Liu S., Shoucair S., Ding N., Ji Y., Zhang J., Wang D., Kuang T., Xu X. (2022). Prognostic Value of Tumor-Associated N1/N2 Neutrophil Plasticity in Patients Following Radical Resection of Pancreas Ductal Adenocarcinoma. J. Immunother. Cancer.

[33] Ng M.S.F., Kwok I., Tan L., Shi C., Cerezo-Wallis D., Tan Y., Leong K., Calvo G.F., Yang K., Zhang Y. (2024). Deterministic Reprogramming of Neutrophils within Tumors. Science.

[34] Mihaila A.C., Ciortan L., Macarie R.D., Vadana M., Cecoltan S., Preda M.B., Hudita A., Gan A.-M., Jakobsson G., Tucureanu M.M. (2021). Transcriptional Profiling and Functional Analysis of N1/N2 Neutrophils Reveal an Immunomodulatory Effect of S100A9-Blockade on the Pro-Inflammatory N1 Subpopulation. Front. Immunol..

[35] Scapini P., Lapinet-Vera J.A., Gasperini S., Calzetti F., Bazzoni F., Cassatella M.A. (2000). The Neutrophil as a Cellular Source of Chemokines. Immunol. Rev..

[36] Veglia F., Tyurin V.A., Blasi M., De Leo A., Kossenkov A.V., Donthireddy L., To T.K.J., Schug Z., Basu S., Wang F. (2019). Fatty Acid Transport Protein 2 Reprograms Neutrophils in Cancer. Nature.

[37] Hsieh C.-C., Hung C.-H., Chiang M., Tsai Y.-C., He J.-T. (2019). Hepatic Stellate Cells Enhance Liver Cancer Progression by Inducing Myeloid-Derived Suppressor Cells through Interleukin-6 Signaling. Int. J. Mol. Sci..

[38] Wu Y., Zhao Q., Peng C., Sun L., Li X., Kuang D. (2011). Neutrophils Promote Motility of Cancer Cells via a Hyaluronan-mediated TLR4/PI3K Activation Loop. J. Pathol..

[39] He K., Liu X., Hoffman R.D., Shi R., Lv G., Gao J. (2022). G-CSF/GM-CSF-induced Hematopoietic Dysregulation in the Progression of Solid Tumors. FEBS Open Bio.

[40] Chen C.-L., Wang Y., Huang C.-Y., Zhou Z.-Q., Zhao J.-J., Zhang X.-F., Pan Q.-Z., Wu J.-X., Weng D.-S., Tang Y. (2018). IL-17 Induces Antitumor Immunity by Promoting Beneficial Neutrophil Recruitment and Activation in Esophageal Squamous Cell Carcinoma. OncoImmunology.

[41] Fridlender Z.G., Albelda S.M. (2012). Tumor-Associated Neutrophils: Friend or Foe?. Carcinogenesis.

[42] Granot Z., Henke E., Comen E.A., King T.A., Norton L., Benezra R. (2011). Tumor Entrained Neutrophils Inhibit Seeding in the Premetastatic Lung. Cancer Cell.

[43] Mahiddine K., Blaisdell A., Ma S., Créquer-Grandhomme A., Lowell C.A., Erlebacher A. (2019). Relief of Tumor Hypoxia Unleashes the Tumoricidal Potential of Neutrophils. J. Clin. Investig..

[44] Gershkovitz M., Caspi Y., Fainsod-Levi T., Katz B., Michaeli J., Khawaled S., Lev S., Polyansky L., Shaul M.E., Sionov R.V. (2018). TRPM2 Mediates Neutrophil Killing of Disseminated Tumor Cells. Cancer Res..

[45] Cui C., Chakraborty K., Tang X.A., Zhou G., Schoenfelt K.Q., Becker K.M., Hoffman A., Chang Y.-F., Blank A., Reardon C.A. (2021). Neutrophil Elastase Selectively Kills Cancer Cells and Attenuates Tumorigenesis. Cell.

[46] Finisguerra V., Di Conza G., Di Matteo M., Serneels J., Costa S., Thompson A.A.R., Wauters E., Walmsley S., Prenen H., Granot Z. (2015). MET Is Required for the Recruitment of Anti-Tumoural Neutrophils. Nature.

[47] Sun B., Qin W., Song M., Liu L., Yu Y., Qi X., Sun H. (2018). Neutrophil Suppresses Tumor Cell Proliferation via Fas /Fas Ligand Pathway Mediated Cell Cycle Arrested. Int. J. Biol. Sci..

[48] Van Egmond M., Bakema J.E. (2013). Neutrophils as Effector Cells for Antibody-Based Immunotherapy of Cancer. Semin. Cancer Biol..

[49] Hirschhorn D., Budhu S., Kraehenbuehl L., Gigoux M., Schröder D., Chow A., Ricca J.M., Gasmi B., De Henau O., Mangarin L.M.B. (2023). T Cell Immunotherapies Engage Neutrophils to Eliminate Tumor Antigen Escape Variants. Cell.

[50] Singhal S., Bhojnagarwala P.S., O’Brien S., Moon E.K., Garfall A.L., Rao A.S., Quatromoni J.G., Stephen T.L., Litzky L., Deshpande C. (2016). Origin and Role of a Subset of Tumor-Associated Neutrophils with Antigen-Presenting Cell Features in Early-Stage Human Lung Cancer. Cancer Cell.

[51] Pylaeva E., Korschunow G., Spyra I., Bordbari S., Siakaeva E., Ozel I., Domnich M., Squire A., Hasenberg A., Thangavelu K. (2022). During Early Stages of Cancer, Neutrophils Initiate Anti-Tumor Immune Responses in Tumor-Draining Lymph Nodes. Cell Rep..

[52] Gungabeesoon J., Gort-Freitas N.A., Kiss M., Bolli E., Messemaker M., Siwicki M., Hicham M., Bill R., Koch P., Cianciaruso C. (2023). A Neutrophil Response Linked to Tumor Control in Immunotherapy. Cell.

[53] Ponzetta A., Carriero R., Carnevale S., Barbagallo M., Molgora M., Perucchini C., Magrini E., Gianni F., Kunderfranco P., Polentarutti N. (2019). Neutrophils Driving Unconventional T Cells Mediate Resistance against Murine Sarcomas and Selected Human Tumors. Cell.

[54] Najmeh S., Cools-Lartigue J., Rayes R.F., Gowing S., Vourtzoumis P., Bourdeau F., Giannias B., Berube J., Rousseau S., Ferri L.E. (2017). Neutrophil Extracellular Traps Sequester Circulating Tumor Cells via Β1-Integrin Mediated Interactions: NETs Sequester CTCs via Integrin Β1. Int. J. Cancer.

[55] Sinha P., Clements V.K., Bunt S.K., Albelda S.M., Ostrand-Rosenberg S. (2007). Cross-Talk between Myeloid-Derived Suppressor Cells and Macrophages Subverts Tumor Immunity toward a Type 2 Response. J. Immunol..

[56] Erdman S.E., Rao V.P., Poutahidis T., Rogers A.B., Taylor C.L., Jackson E.A., Ge Z., Lee C.W., Schauer D.B., Wogan G.N. (2009). Nitric Oxide and TNF-α Trigger Colonic Inflammation and Carcinogenesis in Helicobacter Hepaticus -Infected, Rag2 -Deficient Mice. Proc. Natl. Acad. Sci. USA.

[57] Canli Ö., Nicolas A.M., Gupta J., Finkelmeier F., Goncharova O., Pesic M., Neumann T., Horst D., Löwer M., Sahin U. (2017). Myeloid Cell-Derived Reactive Oxygen Species Induce Epithelial Mutagenesis. Cancer Cell.

[58] Butin-Israeli V., Bui T.M., Wiesolek H.L., Mascarenhas L., Lee J.J., Mehl L.C., Knutson K.R., Adam S.A., Goldman R.D., Beyder A. (2019). Neutrophil-Induced Genomic Instability Impedes Resolution of Inflammation and Wound Healing. J. Clin. Investig..

[59] Houghton A.M., Rzymkiewicz D.M., Ji H., Gregory A.D., Egea E.E., Metz H.E., Stolz D.B., Land S.R., Marconcini L.A., Kliment C.R. (2010). Neutrophil Elastase–Mediated Degradation of IRS-1 Accelerates Lung Tumor Growth. Nat. Med..

[60] Antonio N., Bønnelykke-Behrndtz M.L., Ward L.C., Collin J., Christensen I.J., Steiniche T., Schmidt H., Feng Y., Martin P. (2015). The Wound Inflammatory Response Exacerbates Growth of Pre-neoplastic Cells and Progression to Cancer. EMBO J..

[61] Bancaro N., Calì B., Troiani M., Elia A.R., Arzola R.A., Attanasio G., Lai P., Crespo M., Gurel B., Pereira R. (2023). Apolipoprotein E Induces Pathogenic Senescent-like Myeloid Cells in Prostate Cancer. Cancer Cell.

[62] Di Mitri D., Toso A., Chen J.J., Sarti M., Pinton S., Jost T.R., D’Antuono R., Montani E., Garcia-Escudero R., Guccini I. (2014). Tumour-Infiltrating Gr-1+ Myeloid Cells Antagonize Senescence in Cancer. Nature.

[63] Denk D., Greten F.R. (2022). Inflammation: The Incubator of the Tumor Microenvironment. Trends Cancer.

[64] Boone B.A., Orlichenko L., Schapiro N.E., Loughran P., Gianfrate G.C., Ellis J.T., Singhi A.D., Kang R., Tang D., Lotze M.T. (2015). The Receptor for Advanced Glycation End Products (RAGE) Enhances Autophagy and Neutrophil Extracellular Traps in Pancreatic Cancer. Cancer Gene Ther..

[65] Munir H., Jones J.O., Janowitz T., Hoffmann M., Euler M., Martins C.P., Welsh S.J., Shields J.D. (2021). Stromal-Driven and Amyloid β-Dependent Induction of Neutrophil Extracellular Traps Modulates Tumor Growth. Nat. Commun..

[66] Wang M., Yu F., Zhang Y., Li P. (2024). Novel Insights into Notch Signaling in Tumor Immunity: Potential Targets for Cancer Immunotherapy. Front. Immunol..

[67] Zha C., Meng X., Li L., Mi S., Qian D., Li Z., Wu P., Hu S., Zhao S., Cai J. (2020). Neutrophil Extracellular Traps Mediate the Crosstalk between Glioma Progression and the Tumor Microenvironment via the HMGB1/RAGE/IL-8 Axis. Cancer Biol. Med..

[68] Shojaei F., Singh M., Thompson J.D., Ferrara N. (2008). Role of Bv8 in Neutrophil-Dependent Angiogenesis in a Transgenic Model of Cancer Progression. Proc. Natl. Acad. Sci. USA.

[69] Kusumanto Y.H., Dam W.A., Hospers G.A.P., Meijer C., Mulder N.H. (2003). Platelets and Granulocytes, in Particular the Neutrophils, Form Important Compartments for Circulating Vascular Endothelial Growth Factor. Angiogenesis.

[70] Gordon-Weeks A.N., Lim S.Y., Yuzhalin A.E., Jones K., Markelc B., Kim K.J., Buzzelli J.N., Fokas E., Cao Y., Smart S. (2017). Neutrophils Promote Hepatic Metastasis Growth through Fibroblast Growth Factor 2–Dependent Angiogenesis in Mice. Hepatology.

[71] Yang L., DeBusk L.M., Fukuda K., Fingleton B., Green-Jarvis B., Shyr Y., Matrisian L.M., Carbone D.P., Lin P.C. (2004). Expansion of Myeloid Immune Suppressor Gr+CD11b+ Cells in Tumor-Bearing Host Directly Promotes Tumor Angiogenesis. Cancer Cell.

[72] Queen M.M., Ryan R.E., Holzer R.G., Keller-Peck C.R., Jorcyk C.L. (2005). Breast Cancer Cells Stimulate Neutrophils to Produce Oncostatin M: Potential Implications for Tumor Progression. Cancer Res..

[73] Mittal V. (2018). Epithelial Mesenchymal Transition in Tumor Metastasis. Annu. Rev. Pathol. Mech. Dis..

[74] Li S., Cong X., Gao H., Lan X., Li Z., Wang W., Song S., Wang Y., Li C., Zhang H. (2019). Tumor-Associated Neutrophils Induce EMT by IL-17a to Promote Migration and Invasion in Gastric Cancer Cells. J. Exp. Clin. Cancer Res..

[75] Große-Steffen T., Giese T., Giese N., Longerich T., Schirmacher P., Hänsch G.M., Gaida M.M. (2012). Epithelial-to-Mesenchymal Transition in Pancreatic Ductal Adenocarcinoma and Pancreatic Tumor Cell Lines: The Role of Neutrophils and Neutrophil-Derived Elastase. Clin. Dev. Immunol..

[76] Albrengues J., Shields M.A., Ng D., Park C.G., Ambrico A., Poindexter M.E., Upadhyay P., Uyeminami D.L., Pommier A., Küttner V. (2018). Neutrophil Extracellular Traps Produced during Inflammation Awaken Dormant Cancer Cells in Mice. Science.

[77] Huh S.J., Liang S., Sharma A., Dong C., Robertson G.P. (2010). Transiently Entrapped Circulating Tumor Cells Interact with Neutrophils to Facilitate Lung Metastasis Development. Cancer Res..

[78] Teijeira Á., Garasa S., Gato M., Alfaro C., Migueliz I., Cirella A., De Andrea C., Ochoa M.C., Otano I., Etxeberria I. (2020). CXCR1 and CXCR2 Chemokine Receptor Agonists Produced by Tumors Induce Neutrophil Extracellular Traps That Interfere with Immune Cytotoxicity. Immunity.

[79] Li P., Lu M., Shi J., Gong Z., Hua L., Li Q., Lim B., Zhang X.H.-F., Chen X., Li S. (2020). Lung Mesenchymal Cells Elicit Lipid Storage in Neutrophils That Fuel Breast Cancer Lung Metastasis. Nat. Immunol..

[80] Sukumar M., Roychoudhuri R., Restifo N.P. (2015). Nutrient Competition: A New Axis of Tumor Immunosuppression. Cell.

[81] Bohn T., Rapp S., Luther N., Klein M., Bruehl T.-J., Kojima N., Aranda Lopez P., Hahlbrock J., Muth S., Endo S. (2018). Tumor Immunoevasion via Acidosis-Dependent Induction of Regulatory Tumor-Associated Macrophages. Nat. Immunol..

[82] Kim R., Hashimoto A., Markosyan N., Tyurin V.A., Tyurina Y.Y., Kar G., Fu S., Sehgal M., Garcia-Gerique L., Kossenkov A. (2022). Ferroptosis of Tumour Neutrophils Causes Immune Suppression in Cancer. Nature.

[83] Jin C., Lagoudas G.K., Zhao C., Bullman S., Bhutkar A., Hu B., Ameh S., Sandel D., Liang X.S., Mazzilli S. (2019). Commensal Microbiota Promote Lung Cancer Development via Γδ T Cells. Cell.

[84] Ou B., Liu Y., Gao Z., Xu J., Yan Y., Li Y., Zhang J. (2022). Senescent Neutrophils-Derived Exosomal piRNA-17560 Promotes Chemoresistance and EMT of Breast Cancer via FTO-Mediated m6A Demethylation. Cell Death Dis..

[85] Zhu Q., Zhang X., Zhang L., Li W., Wu H., Yuan X., Mao F., Wang M., Zhu W., Qian H. (2014). The IL-6-STAT3 Axis Mediates a Reciprocal Crosstalk between Cancer-Derived Mesenchymal Stem Cells and Neutrophils to Synergistically Prompt Gastric Cancer Progression. Cell Death Dis..

[86] He G., Zhang H., Zhou J., Wang B., Chen Y., Kong Y., Xie X., Wang X., Fei R., Wei L. (2015). Peritumoural Neutrophils Negatively Regulate Adaptive Immunity via the PD-L1/PD-1 Signalling Pathway in Hepatocellular Carcinoma. J. Exp. Clin. Cancer Res..

[87] Cheng Y., Li H., Deng Y., Tai Y., Zeng K., Zhang Y., Liu W., Zhang Q., Yang Y. (2018). Cancer-Associated Fibroblasts Induce PDL1+ Neutrophils through the IL6-STAT3 Pathway That Foster Immune Suppression in Hepatocellular Carcinoma. Cell Death Dis..

[88] Shi Y., Zhang J., Mao Z., Jiang H., Liu W., Shi H., Ji R., Xu W., Qian H., Zhang X. (2020). Extracellular Vesicles From Gastric Cancer Cells Induce PD-L1 Expression on Neutrophils to Suppress T-Cell Immunity. Front. Oncol..

[89] Noman M.Z., Desantis G., Janji B., Hasmim M., Karray S., Dessen P., Bronte V., Chouaib S. (2014). PD-L1 Is a Novel Direct Target of HIF-1α, and Its Blockade under Hypoxia Enhanced MDSC-Mediated T Cell Activation. J. Exp. Med..

[90] Kwantwi L.B., Wang S., Zhang W., Peng W., Cai Z., Sheng Y., Xiao H., Wang X., Wu Q. (2021). Tumor-Associated Neutrophils Activated by Tumor-Derived CCL20 (C-C Motif Chemokine Ligand 20) Promote T Cell Immunosuppression via Programmed Death-Ligand 1 (PD-L1) in Breast Cancer. Bioengineered.

[91] Shang A., Wang W., Gu C., Chen C., Zeng B., Yang Y., Ji P., Sun J., Wu J., Lu W. (2019). Long Non-Coding RNA HOTTIP Enhances IL-6 Expression to Potentiate Immune Escape of Ovarian Cancer Cells by Upregulating the Expression of PD-L1 in Neutrophils. J. Exp. Clin. Cancer Res..

[92] Wang T., Zhao Y., Peng L., Chen N., Chen W., Lv Y., Mao F., Zhang J., Cheng P., Teng Y. (2017). Tumour-Activated Neutrophils in Gastric Cancer Foster Immune Suppression and Disease Progression through GM-CSF-PD-L1 Pathway. Gut.

[93] Anand U., Dey A., Chandel A.K.S., Sanyal R., Mishra A., Pandey D.K., De Falco V., Upadhyay A., Kandimalla R., Chaudhary A. (2023). Cancer Chemotherapy and beyond: Current Status, Drug Candidates, Associated Risks and Progress in Targeted Therapeutics. Genes Dis..

[94] Amjad M.T., Chidharla A., Kasi A. (2025). Cancer Chemotherapy. StatPearls.

[95] Chen G.Y., Nuñez G. (2010). Sterile Inflammation: Sensing and Reacting to Damage. Nat. Rev. Immunol..

[96] Greten F.R., Grivennikov S.I. (2019). Inflammation and Cancer: Triggers, Mechanisms, and Consequences. Immunity.

[97] Grivennikov S.I., Greten F.R., Karin M. (2010). Immunity, Inflammation, and Cancer. Cell.

[98] Chen F., Tang H., Cai X., Lin J., Kang R., Tang D., Liu J. (2024). DAMPs in Immunosenescence and Cancer. Semin. Cancer Biol..

[99] Ozga A.J., Chow M.T., Luster A.D. (2021). Chemokines and the Immune Response to Cancer. Immunity.

[100] Liu S., Wu W., Du Y., Yin H., Chen Q., Yu W., Wang W., Yu J., Liu L., Lou W. (2023). The Evolution and Heterogeneity of Neutrophils in Cancers: Origins, Subsets, Functions, Orchestrations and Clinical Applications. Mol. Cancer.

[101] Zhang M., Qin H., Wu Y., Gao Q. (2024). Complex Role of Neutrophils in the Tumor Microenvironment: An Avenue for Novel Immunotherapies. Cancer Biol. Med..

[102] Onier N., Hilpert S., Reveneau S., Arnould L., Saint-Giorgio V., Exbrayat J.M., Jeannin J.F. (1999). Expression of Inducible Nitric Oxide Synthase in Tumors in Relation with Their Regression Induced by Lipid A in Rats. Int. J. Cancer.

[103] Seignez C., Martin A., Rollet C.-E., Racoeur C., Scagliarini A., Jeannin J.-F., Bettaieb A., Paul C. (2014). Senescence of Tumor Cells Induced by Oxaliplatin Increases the Efficiency of a Lipid A Immunotherapy via the Recruitment of Neutrophils. Oncotarget.

[104] Zhou Z., Zhao Y., Chen S., Cui G., Fu W., Li S., Lin X., Hu H. (2022). Cisplatin Promotes the Efficacy of Immune Checkpoint Inhibitor Therapy by Inducing Ferroptosis and Activating Neutrophils. Front. Pharmacol..

[105] Balkwill F. (2009). Tumour Necrosis Factor and Cancer. Nat. Rev. Cancer.

[106] Martin A., Seignez C., Racoeur C., Isambert N., Mabrouk N., Scagliarini A., Reveneau S., Arnould L., Bettaieb A., Jeannin J.-F. (2018). Tumor-Derived Granzyme B-Expressing Neutrophils Acquire Antitumor Potential after Lipid A Treatment. Oncotarget.

[107] Li Y., Wu S., Zhao Y., Dinh T., Jiang D., Selfridge J.E., Myers G., Wang Y., Zhao X., Tomchuck S. (2024). Neutrophil Extracellular Traps Induced by Chemotherapy Inhibit Tumor Growth in Murine Models of Colorectal Cancer. J. Clin. Investig..

[108] Gan Y., Li X., Han S., Liang Q., Ma X., Rong P., Wang W., Li W. (2022). The cGAS/STING Pathway: A Novel Target for Cancer Therapy. Front. Immunol..

[109] Jablonska J., Wu C.-F., Andzinski L., Leschner S., Weiss S. (2014). CXCR2-mediated Tumor-associated Neutrophil Recruitment Is Regulated by IFN-β. Int. J. Cancer.

[110] Sasaki S., Baba T., Muranaka H., Tanabe Y., Takahashi C., Matsugo S., Mukaida N. (2018). Involvement of Prokineticin 2–Expressing Neutrophil Infiltration in 5-Fluorouracil–Induced Aggravation of Breast Cancer Metastasis to Lung. Mol. Cancer Ther..

[111] Kong L., Hu S., Zhao Y., Huang Y., Xiang X., Yu Y., Mao X., Xie K., Zhu X., Xu P. (2025). Neutrophil Extracellular Traps Induced by Neoadjuvant Chemotherapy of Breast Cancer Promotes Vascular Endothelial Damage. Breast Cancer Res..

[112] Martin K.R., Wong H.L., Witko-Sarsat V., Wicks I.P. (2021). G-CSF—A Double Edge Sword in Neutrophil Mediated Immunity. Semin. Immunol..

[113] Mehta H.M., Malandra M., Corey S.J. (2015). G-CSF and GM-CSF in Neutropenia. J. Immunol..

[114] Panopoulos A.D., Watowich S.S. (2008). Granulocyte Colony-Stimulating Factor: Molecular Mechanisms of Action during Steady State and ‘Emergency’ Hematopoiesis. Cytokine.

[115] Demers M., Krause D.S., Schatzberg D., Martinod K., Voorhees J.R., Fuchs T.A., Scadden D.T., Wagner D.D. (2012). Cancers Predispose Neutrophils to Release Extracellular DNA Traps That Contribute to Cancer-Associated Thrombosis. Proc. Natl. Acad. Sci. USA.

[116] Park J., Wysocki R.W., Amoozgar Z., Maiorino L., Fein M.R., Jorns J., Schott A.F., Kinugasa-Katayama Y., Lee Y., Won N.H. (2016). Cancer Cells Induce Metastasis-Supporting Neutrophil Extracellular DNA Traps. Sci. Transl. Med..

[117] Zhao Y., Feng X., Chen Y., Selfridge J.E., Gorityala S., Du Z., Wang J.M., Hao Y., Cioffi G., Conlon R.A. (2020). 5-Fluorouracil Enhances the Antitumor Activity of the Glutaminase Inhibitor CB-839 against PIK3CA -Mutant Colorectal Cancers. Cancer Res..

[118] Wang C.-Y., Lin T.-T., Hu L., Xu C.-J., Hu F., Wan L., Yang X., Wu X.-F., Zhang X.-T., Li Y. (2023). Neutrophil Extracellular Traps as a Unique Target in the Treatment of Chemotherapy-Induced Peripheral Neuropathy. eBioMedicine.

[119] Rout P., Reynolds S.B., Zito P.M. (2024). Neutropenia. StatPearls.

[120] Lustberg M.B. (2012). Management of Neutropenia in Cancer Patients. Clin. Adv. Hematol. Oncol. HO.

[121] Liu X., Arfman T., Wichapong K., Reutelingsperger C.P.M., Voorberg J., Nicolaes G.A.F. (2021). PAD4 Takes Charge during Neutrophil Activation: Impact of PAD4 Mediated NET Formation on Immune-mediated Disease. J. Thromb. Haemost..

[122] Vogel B., Shinagawa H., Hofmann U., Ertl G., Frantz S. (2015). Acute DNase1 Treatment Improves Left Ventricular Remodeling after Myocardial Infarction by Disruption of Free Chromatin. Basic Res. Cardiol..

[123] Fauvre A., Ursino C., Garambois V., Culerier E., Milazzo L.-A., Vezzio-Vié N., Jeanson L., Marchive C., Andrade A.F., Combes E. (2025). Oxaliplatin, ATR Inhibitor and Anti-PD-1 Antibody Combination Therapy Controls Colon Carcinoma Growth, Induces Local and Systemic Changes in the Immune Compartment, and Protects against Tumor Rechallenge in Mice. J. Immunother. Cancer.

[124] Wheldon T.E., O’Donoghue J.A. (1990). The Radiobiology of Targeted Radiotherapy. Int. J. Radiat. Biol..

[125] Jackson S.P., Bartek J. (2009). The DNA-Damage Response in Human Biology and Disease. Nature.

[126] Maier P., Hartmann L., Wenz F., Herskind C. (2016). Cellular Pathways in Response to Ionizing Radiation and Their Targetability for Tumor Radiosensitization. Int. J. Mol. Sci..

[127] Begg A.C., Stewart F.A., Vens C. (2011). Strategies to Improve Radiotherapy with Targeted Drugs. Nat. Rev. Cancer.

[128] Baskar R., Lee K.A., Yeo R., Yeoh K.-W. (2012). Cancer and Radiation Therapy: Current Advances and Future Directions. Int. J. Med. Sci..

[129] Maani E.V., Maani C.V. (2022). Radiation Therapy. StatPearls.

[130] Raymakers L., Demmers T.J., Meijer G.J., Molenaar I.Q., Van Santvoort H.C., Intven M.P.W., Leusen J.H.W., Olofsen P.A., Daamen L.A. (2024). The Effect of Radiation Treatment of Solid Tumors on Neutrophil Infiltration and Function: A Systematic Review. Int. J. Radiat. Oncol..

[131] Zhang F., Mulvaney O., Salcedo E., Manna S., Zhu J.Z., Wang T., Ahn C., Pop L.M., Hannan R. (2023). Radiation-Induced Innate Neutrophil Response in Tumor Is Mediated by the CXCLs/CXCR2 Axis. Cancers.

[132] Chao T., Furth E.E., Vonderheide R.H. (2016). CXCR2-Dependent Accumulation of Tumor-Associated Neutrophils Regulates T-Cell Immunity in Pancreatic Ductal Adenocarcinoma. Cancer Immunol. Res..

[133] Guan L., Nambiar D.K., Cao H., Viswanathan V., Kwok S., Hui A.B., Hou Y., Hildebrand R., Von Eyben R., Holmes B.J. (2023). NFE2L2 Mutations Enhance Radioresistance in Head and Neck Cancer by Modulating Intratumoral Myeloid Cells. Cancer Res..

[134] Trappetti V., Potez M., Fernandez-Palomo C., Volarevic V., Shintani N., Pellicioli P., Ernst A., Haberthür D., Fazzari J.M., Krisch M. (2022). Microbeam Radiation Therapy Controls Local Growth of Radioresistant Melanoma and Treats Out-of-Field Locoregional Metastasis. Int. J. Radiat. Oncol..

[135] Yang X., Lu Y., Hang J., Zhang J., Zhang T., Huo Y., Liu J., Lai S., Luo D., Wang L. (2020). Lactate-Modulated Immunosuppression of Myeloid-Derived Suppressor Cells Contributes to the Radioresistance of Pancreatic Cancer. Cancer Immunol. Res..

[136] Oweida A.J., Mueller A.C., Piper M., Milner D., Van Court B., Bhatia S., Phan A., Bickett T., Jordan K., Proia T. (2021). Response to Radiotherapy in Pancreatic Ductal Adenocarcinoma Is Enhanced by Inhibition of Myeloid-Derived Suppressor Cells Using STAT3 Anti-Sense Oligonucleotide. Cancer Immunol. Immunother..

[137] Liu Q., Hao Y., Du R., Hu D., Xie J., Zhang J., Deng G., Liang N., Tian T., Käsmann L. (2021). Radiotherapy Programs Neutrophils to an Antitumor Phenotype by Inducing Mesenchymal-Epithelial Transition. Transl. Lung Cancer Res..

[138] Takeshima T., Pop L.M., Laine A., Iyengar P., Vitetta E.S., Hannan R. (2016). Key Role for Neutrophils in Radiation-Induced Antitumor Immune Responses: Potentiation with G-CSF. Proc. Natl. Acad. Sci. USA.

[139] Bian Z., Shi L., Kidder K., Zen K., Garnett-Benson C., Liu Y. (2021). Intratumoral SIRPα-Deficient Macrophages Activate Tumor Antigen-Specific Cytotoxic T Cells under Radiotherapy. Nat. Commun..

[140] Wu C.-T., Chen M.-F., Chen W.-C., Hsieh C.-C. (2013). The Role of IL-6 in the Radiation Response of Prostate Cancer. Radiat. Oncol..

[141] Nolan E., Bridgeman V.L., Ombrato L., Karoutas A., Rabas N., Sewnath C.A.N., Vasquez M., Rodrigues F.S., Horswell S., Faull P. (2022). Radiation Exposure Elicits a Neutrophil-Driven Response in Healthy Lung Tissue That Enhances Metastatic Colonization. Nat. Cancer.

[142] Wisdom A.J., Hong C.S., Lin A.J., Xiang Y., Cooper D.E., Zhang J., Xu E.S., Kuo H.-C., Mowery Y.M., Carpenter D.J. (2019). Neutrophils Promote Tumor Resistance to Radiation Therapy. Proc. Natl. Acad. Sci. USA.

[143] Shinde-Jadhav S., Mansure J.J., Rayes R.F., Marcq G., Ayoub M., Skowronski R., Kool R., Bourdeau F., Brimo F., Spicer J. (2021). Role of Neutrophil Extracellular Traps in Radiation Resistance of Invasive Bladder Cancer. Nat. Commun..

[144] Teijeira A., Garasa S., Ochoa M.C., Sanchez-Gregorio S., Gomis G., Luri-Rey C., Martinez-Monge R., Pinci B., Valencia K., Palencia B. (2024). Low-Dose Ionizing γ-Radiation Elicits the Extrusion of Neutrophil Extracellular Traps. Clin. Cancer Res..

[145] Zhang J., Zhang L., Yang Y., Liu Q., Ma H., Huang A., Zhao Y., Xia Z., Liu T., Wu G. (2021). Polymorphonuclear-MDSCs Facilitate Tumor Regrowth After Radiation by Suppressing CD8+ T Cells. Int. J. Radiat. Oncol..

[146] Leonard W., Dufait I., Schwarze J.K., Law K., Engels B., Jiang H., Van Den Berge D., Gevaert T., Storme G., Verovski V. (2016). Myeloid-Derived Suppressor Cells Reveal Radioprotective Properties through Arginase-Induced l-Arginine Depletion. Radiother. Oncol..

[147] Zheng Z., Su J., Bao X., Wang H., Bian C., Zhao Q., Jiang X. (2023). Mechanisms and Applications of Radiation-Induced Oxidative Stress in Regulating Cancer Immunotherapy. Front. Immunol..

[148] Haidenberger A., Hengster P., Kunc M., Micke O., Wolfgruber T., Auer T., Lukas P., DeVries A. (2003). Influence of Fractionated Irradiation on Neutrophilic Granulocyte Function. Strahlenther. Onkol..

[149] Schernberg A., Blanchard P., Chargari C., Deutsch E. (2017). Neutrophils, a Candidate Biomarker and Target for Radiation Therapy?. Acta Oncol. Stockh. Swed..

[150] Fu S.-Y., Chen F.-H., Wang C.-C., Yu C.-F., Chiang C.-S., Hong J.-H. (2021). Role of Myeloid-Derived Suppressor Cells in High-Dose-Irradiated TRAMP-C1 Tumors: A Therapeutic Target and an Index for Assessing Tumor Microenvironment. Int. J. Radiat. Oncol..

[151] Jiménez-Cortegana C., Galassi C., Klapp V., Gabrilovich D.I., Galluzzi L. (2022). Myeloid-Derived Suppressor Cells and Radiotherapy. Cancer Immunol. Res..

[152] Yang Y., Li C., Liu T., Dai X., Bazhin A.V. (2020). Myeloid-Derived Suppressor Cells in Tumors: From Mechanisms to Antigen Specificity and Microenvironmental Regulation. Front. Immunol..

[153] Liu M., Wang X., Wang L., Ma X., Gong Z., Zhang S., Li Y. (2018). Targeting the IDO1 Pathway in Cancer: From Bench to Bedside. J. Hematol. Oncol..

[154] Hu J., Pan M., Wang Y., Zhu Y., Wang M. (2023). Functional Plasticity of Neutrophils after Low- or High-Dose Irradiation in Cancer Treatment—A Mini Review. Front. Immunol..

[155] Kalos M., June C.H. (2013). Adoptive T Cell Transfer for Cancer Immunotherapy in the Era of Synthetic Biology. Immunity.

[156] June C.H., O’Connor R.S., Kawalekar O.U., Ghassemi S., Milone M.C. (2018). CAR T Cell Immunotherapy for Human Cancer. Science.

[157] Cheng M., Chen Y., Xiao W., Sun R., Tian Z. (2013). NK Cell-Based Immunotherapy for Malignant Diseases. Cell. Mol. Immunol..

[158] Anderson N.R., Minutolo N.G., Gill S., Klichinsky M. (2021). Macrophage-Based Approaches for Cancer Immunotherapy. Cancer Res..

[159] Tang H., Qiao J., Fu Y.-X. (2016). Immunotherapy and Tumor Microenvironment. Cancer Lett..

[160] Zhang J., Shi Z., Xu X., Yu Z., Mi J. (2019). The Influence of Microenvironment on Tumor Immunotherapy. FEBS J..

[161] Yao J., Ji L., Wang G., Ding J. (2025). Effect of Neutrophils on Tumor Immunity and Immunotherapy Resistance with Underlying Mechanisms. Cancer Commun. Lond. Engl..

[162] Zhang M., Long X., Xiao Y., Jin J., Chen C., Meng J., Liu W., Liu A., Chen L. (2023). Assessment and Predictive Ability of the Absolute Neutrophil Count in Peripheral Blood for in Vivo CAR T Cells Expansion and CRS. J. Immunother. Cancer.

[163] Xiao X., Huang S., Chen S., Wang Y., Sun Q., Xu X., Li Y. (2021). Mechanisms of Cytokine Release Syndrome and Neurotoxicity of CAR T-Cell Therapy and Associated Prevention and Management Strategies. J. Exp. Clin. Cancer Res..

[164] Hao Z., Li R., Meng L., Han Z., Hong Z. (2020). Macrophage, the Potential Key Mediator in CAR-T Related CRS. Exp. Hematol. Oncol..

[165] Yang S., Xu J., Dai Y., Jin S., Sun Y., Li J., Liu C., Ma X., Chen Z., Chen L. (2024). Neutrophil Activation and Clonal CAR-T Re-Expansion Underpinning Cytokine Release Syndrome during Ciltacabtagene Autoleucel Therapy in Multiple Myeloma. Nat. Commun..

[166] Zhu J., Zhou J., Liang X., An F., Ding Y., Jiao X., Xiao M., Wu F., Li Y., Xiao H. (2025). Elevated CD10- Neutrophils Correlate with Non-Response and Poor Prognosis of CD19 CAR T-Cell Therapy for B-Cell Acute Lymphoblastic Leukemia. BMC Med..

[167] Harris J.D., Chang Y., Syahirah R., Lian X.L., Deng Q., Bao X. (2023). Engineered Anti-Prostate Cancer CAR-Neutrophils from Human Pluripotent Stem Cells. J. Immunol. Regen. Med..

[168] Chang Y., Syahirah R., Wang X., Jin G., Torregrosa-Allen S., Elzey B.D., Hummel S.N., Wang T., Li C., Lian X. (2022). Engineering Chimeric Antigen Receptor Neutrophils from Human Pluripotent Stem Cells for Targeted Cancer Immunotherapy. Cell Rep..

[169] Majumder A., Jung H.S., Zhang J., Suknuntha K., Thomson J., Slukvin I. (2022). 356 Generation of GD2-CAR Neutrophils from hPSCs for Targeted Cancer Immunotherapy of Solid Tumors. J. Immunother. Cancer.

[170] Chang Y., Cai X., Syahirah R., Yao Y., Xu Y., Jin G., Bhute V.J., Torregrosa-Allen S., Elzey B.D., Won Y.-Y. (2023). CAR-Neutrophil Mediated Delivery of Tumor-Microenvironment Responsive Nanodrugs for Glioblastoma Chemo-Immunotherapy. Nat. Commun..

[171] Harrington K., Freeman D.J., Kelly B., Harper J., Soria J.-C. (2019). Optimizing Oncolytic Virotherapy in Cancer Treatment. Nat. Rev. Drug Discov..

[172] Varghese S., Rabkin S.D. (2002). Oncolytic Herpes Simplex Virus Vectors for Cancer Virotherapy. Cancer Gene Ther..

[173] Kirn D. (2001). Oncolytic Virotherapy for Cancer with the Adenovirus Dl1520 (Onyx-015): Results of Phase I and II Trials. Expert Opin. Biol. Ther..

[174] Deng L., Fan J., Ding Y., Zhang J., Zhou B., Zhang Y., Huang B., Hu Z. (2019). Oncolytic Cancer Therapy with a Vaccinia Virus Strain. Oncol. Rep..

[175] Zhang J., Chen J., Lin K. (2024). Immunogenic Cell Death-Based Oncolytic Virus Therapy: A Sharp Sword of Tumor Immunotherapy. Eur. J. Pharmacol..

[176] DePeaux K., Delgoffe G.M. (2024). Integrating Innate and Adaptive Immunity in Oncolytic Virus Therapy. Trends Cancer.

[177] Zhou D., Zhang C., Sun J., Yuan M. (2024). Neutrophils in Oncolytic Virus Immunotherapy. Front. Immunol..

[178] Mealiea D., McCart J.A. (2022). Cutting Both Ways: The Innate Immune Response to Oncolytic Virotherapy. Cancer Gene Ther..

[179] Dai W., Tian R., Yu L., Bian S., Chen Y., Yin B., Luan Y., Chen S., Fan Z., Yan R. (2024). Overcoming Therapeutic Resistance in Oncolytic Herpes Virotherapy by Targeting IGF2BP3-Induced NETosis in Malignant Glioma. Nat. Commun..

[180] Zhou D., Xu W., Ding X., Guo H., Wang J., Zhao G., Zhang C., Zhang Z., Wang Z., Wang P. (2024). Transient Inhibition of Neutrophil Functions Enhances the Antitumor Effect of Intravenously Delivered Oncolytic Vaccinia Virus. Cancer Sci..

[181] Minott J.A., van Vloten J.P., Chan L., Mehrani Y., Bridle B.W., Karimi K. (2022). The Role of Neutrophils in Oncolytic Orf Virus-Mediated Cancer Immunotherapy. Cells.

[182] Staedtke V., Sun N., Bai R. (2024). Hypoxia-Targeting Bacteria in Cancer Therapy. Semin. Cancer Biol..

[183] Theys J., Patterson A.V., Mowday A.M. (2024). Clostridium Bacteria: Harnessing Tumour Necrosis for Targeted Gene Delivery. Mol. Diagn. Ther..

[184] Staedtke V., Roberts N.J., Bai R.-Y., Zhou S. (2016). Clostridium Novyi-NT in Cancer Therapy. Genes Dis..

[185] Staedtke V., Bai R.-Y., Sun W., Huang J., Kibler K.K., Tyler B.M., Gallia G.L., Kinzler K., Vogelstein B., Zhou S. (2015). Clostridium Novyi -NT Can Cause Regression of Orthotopically Implanted Glioblastomas in Rats. Oncotarget.

[186] Janku F., Zhang H.H., Pezeshki A., Goel S., Murthy R., Wang-Gillam A., Shepard D.R., Helgason T., Masters T., Hong D.S. (2021). Intratumoral Injection of Clostridium Novyi -NT Spores in Patients with Treatment-Refractory Advanced Solid Tumors. Clin. Cancer Res..

[187] Bettegowda C., Huang X., Lin J., Cheong I., Kohli M., Szabo S.A., Zhang X., Diaz L.A., Velculescu V.E., Parmigiani G. (2006). The Genome and Transcriptomes of the Anti-Tumor Agent Clostridium Novyi-NT. Nat. Biotechnol..

[188] Zwagerman N.T., Friedlander R.M., Monaco E.A. (2014). Intratumoral Clostridium Novyi as a Potential Treatment for Solid Necrotic Brain Tumors. Neurosurgery.

[189] Staedtke V., Gray-Bethke T., Liu G., Liapi E., Riggins G.J., Bai R.-Y. (2022). Neutrophil Depletion Enhanced the Clostridium Novyi-NT Therapy in Mouse and Rabbit Tumor Models. Neuro-Oncol. Adv..

[190] Coffelt S.B., Wellenstein M.D., de Visser K.E. (2016). Neutrophils in Cancer: Neutral No More. Nat. Rev. Cancer.

[191] Burnette B.C., Liang H., Lee Y., Chlewicki L., Khodarev N.N., Weichselbaum R.R., Fu Y.-X., Auh S.L. (2011). The Efficacy of Radiotherapy Relies upon Induction of Type i Interferon-Dependent Innate and Adaptive Immunity. Cancer Res..

[192] Xie X., Shi Q., Wu P., Zhang X., Kambara H., Su J., Yu H., Park S.-Y., Guo R., Ren Q. (2020). Single-Cell Transcriptome Profiling Reveals Neutrophil Heterogeneity in Homeostasis and Infection. Nat. Immunol..

[193] Wigerblad G., Cao Q., Brooks S., Naz F., Gadkari M., Jiang K., Gupta S., O’Neil L., Dell’Orso S., Kaplan M.J. (2022). Single-Cell Analysis Reveals the Range of Transcriptional States of Circulating Human Neutrophils. J. Immunol..

[194] LaSalle T.J., Gonye A.L.K., Freeman S.S., Kaplonek P., Gushterova I., Kays K.R., Manakongtreecheep K., Tantivit J., Rojas-Lopez M., Russo B.C. (2022). Longitudinal Characterization of Circulating Neutrophils Uncovers Phenotypes Associated with Severity in Hospitalized COVID-19 Patients. Cell Rep. Med..

[195] Chen Z., Li W., Tang Y., Zhou P., He Q., Deng Z. (2024). The Neutrophil-Lymphocyte Ratio Predicts All-Cause and Cardiovascular Mortality among United States Adults with COPD: Results from NHANES 1999–2018. Front. Med..

[196] Xiao M., Zhou J., Zhang W., Ding Y., Guo J., Liang X., Zhu J., Jiao X., Zhai Z., Wang H. (2024). Association of Immunosuppressive CD45^+^CD33^+^CD14^−^CD10^−^HLA-DR^−/low^ Neutrophils with Poor Prognosis in Patients with Lymphoma and Their Expansion and Activation through STAT3/Arginase-1 Pathway in Vitro. Cytojournal.

[197] Liu W., Zhang K., Chen S., Wang X., Yu W. (2024). The Pretreatment Neutrophil-to-Lymphocyte Ratio as a Near-Term Prognostic Indicator in Patients with Locally Advanced Hepatocellular Carcinoma Treated with Hepatic Arterial Infusion Chemotherapy: A Propensity Score Matching Cohort Study. Br. J. Hosp. Med..

[198] Mi H., Wei W., Zhang D., Liang H., Yue C., Xu J. (2024). Neutrophil-to-Lymphocyte Ratio, Platelet-to-Lymphocyte Ratio, and Prognostic Nutritional Index as Prognostic Markers for Lung Carcinoma. Br. J. Hosp. Med..

